# Associations of Blood and Urinary Heavy Metals with Stress Urinary Incontinence Risk Among Adults in NHANES, 2003–2018

**DOI:** 10.1007/s12011-024-04264-8

**Published:** 2024-06-17

**Authors:** Maoling Fu, Zifan Zhu, Yechen Xiang, Qiaoyue Yang, Quan Yuan, Xinyu Li, Genzhen Yu

**Affiliations:** 1 Department of Nursing, Tongji Hospital, Tongji Medical College, Huazhong University of Science and Technology, 1095 Jiefang Road, Wuhan, 430030 Hubei China; 2 School of Nursing, Tongji Medical College, Huazhong University of Science and Technology, 13 Aviation Road, Wuhan, 430030 Hubei China; 3 School of Mental Health and Psychological Science, Anhui Medical University, 81 Meishan Road, Hefei, 230032 Anhui China; 4 Department of Urology, Hunan University of Medicine General Hospital, Hunan University of Medicine, 370 Jinxi South Road, Huaihua, 418000 Hunan China

**Keywords:** Heavy metals, Stress urinary incontinence, Mixture, Co-exposure, NHANES

## Abstract

People come into contact with heavy metals in various ways in their daily lives. Accumulating evidence shows that toxic metal exposure is hazardous to human health. However, limited information is available regarding the impact of metal mixtures on stress urinary incontinence (SUI). Therefore, we used data from 10,622 adults from the 2003–2018 National Health and Nutrition Examination Survey (NHANES) to investigate the independent and comprehensive association between heavy metal co-exposure and SUI. Among them, 2455 (23.1%) had been diagnosed with SUI, while the rest had no SUI. We evaluated the independent and combined associations of 3 blood metals and 10 urinary metals with SUI risk, along with subgroup analyses according to age and gender. In the single-exposure model, blood cadmium (Cd), lead (Pb), mercury (Hg), urinary Cd, Pb, and cesium (Cs) were found to be positively connected with SUI risk. Moreover, weighted quantile sum (WQS) regression, quantile-based g-computation (qgcomp), and Bayesian kernel machine regression (BKMR) consistently demonstrated blood and urinary metal–mixed exposure were positively associated with the risk of SUI, and emphasized that blood Pb and Cd and urinary Cd and Cs were the main positive drivers, respectively. This association was more pronounced in the young and middle-aged group (20–59 years old) and the female group. Nevertheless, further research is necessary to validate these significant findings.

**Supplementary Information:**

The online version contains supplementary material available at 10.1007/s12011-024-04264-8.

## Introduction

The International Continence Society defines urinary incontinence (UI), a common urological condition, as the involuntary outflow of urine under any circumstance [1]. The prevalence of UI increases with age, affecting nearly 50% of adult women worldwide [2–4]. For men, UI is approximately half as common as in women of all ages, with an overall prevalence of 11.5% to 19.3% [5, 6]. UI is divided into three main types: stress UI (SUI, urine involuntary overflow caused by increased abdominal pressure such as sneezing, coughing, laughing, or exercising), urge UI (UUI, involuntary leakage of urine, which can be either accompanied by or directly preceded by urgency), and mixed UI (MUI, any combination of SUI and UUI) [7]. SUI is the most common of these three types, accounting for approximately 31–50% of total UI morbidity [8–10]. Despite the high prevalence of SUI, it remains under-diagnosed and under-treated. Only 25% of affected patients seek care, and much less than 1/2 of them acquire treatment [4, 11]. Although SUI is a non-fatal disease, due to the leakage of urine and body odor caused by the disease, patients often show signs of depression, low self-esteem, and negativity, which puts a great burden on their life, physical, and mental health, as well as their daily social activities [2, 12]. In addition, SUI places a tremendous financial burden on both individuals and national healthcare systems. The US Treasury spends $12 billion annually on treatment for women with SUI alone [13, 14].

SUI has now become a global medical and public health problem, and strengthening research on its etiology has great social significance and clinical value. There is a growing consensus that the impact of environmental factors on disease cannot be ignored. The environment is continually exposed to an extensive variety of heavy metals from anthropogenic sources and natural [15]. The presence of these heavy metals in the surroundings has many adverse consequences on humans and animals. Major human diseases suspected to be caused by exposure to heavy metals include cancers, metabolic syndrome, birth and immune system defects, intellectual retardation, immunotoxicity, and specific organ dysfunction [16–21]. Recent findings from experimental studies show that heavy metal–induced oxidative stress (OS) may also lead to lower urinary tract symptoms (LUTS) and is associated with the development of SUI [22–24]. However, population-based studies on the effects of heavy metals on SUI are limited [25, 26]. Moreover, the function of metals in disease normally relies on their interplay and cooperation with each other. Individual metals cannot thoroughly explain the occurrence and progression of disease. Therefore, exploring the joint effect of metals on SUI is valuable.

For this research, we executed a cross-sectional investigation utilizing the US national population data acquired from the National Health and Nutrition Examination Survey (NHANES) spanning from 2003 to 2018. Five statistical analysis methods assessed the associations of 13 blood and urinary metals and mixtures with SUI risk. Of these, multivariate logistic regression was used to analyze the effects of single metals, and the effects of metals co-exposure were analyzed using weighted quantile sum (WQS) regression, quantile-based g-computation (qgcomp), and Bayesian kernel machine regression (BKMR) models. Furthermore, restricted cubic spline (RCS) analysis was applied to describe the dose–response relationship between heavy metals and SUI. The results from our research offer the latest epidemiological evidence for the association between heavy metals and SUI risk, thereby contributing to the identification of SUI risk factors.

## Methods

### Study Design and Population

NHANES is a periodic national cross-sectional survey that is performed every 2 years to assess the health and nutritional status of non-institutionalized civilians in the United States. The survey endeavor received the approval of the National Center for Health Statistics Ethics Review Board, and all participants submitted informed consent. Detailed information regarding the study protocol or result can be accessed on the Centers for Disease Control and Prevention official website. For this particular investigation, we acquired data from a total of eight NHANES cycles spanning from 2003 to 2018. Participants refusing to answer or not knowing if they had SUI, and lack of data on 13 heavy metals and covariates, were excluded. Collectively, the study involved the recruitment of a total of 10,622 participants, including 2455 patients diagnosed with SUI (Fig. S1).

### Assessment of SUI

The outcomes in the current analysis were SUI. Professional diagnosis and data collection were conducted through a survey questionnaire. The assessment of SUI, performed using this self-reported questionnaire, has been established as a reliable and efficient method [27]. Briefly, participants were asked the question, “Have you leaked or lost control of even a small amount of urine with an activity like coughing, lifting, or exercising?” In this study, those who responded in the affirmative were categorized as people with SUI.

### Metals Measurement

Samples of whole blood and urine underwent processing, storage, and delivery to the Division of Environmental Health Laboratory Science. The levels of blood cadmium (Cd), lead (Pb), mercury (Hg) and urinary barium (Ba), Cd, cobalt (Co), cesium (Cs), molybdenum (Mo), Pb, antimony (Sb), thallium (TI), tungsten (Tu), and arsenic (As) were determined using inductively coupled plasma mass spectrometry (ICP-MS). The ICP ionization source facilitated the entry of the liquid sample into the mass spectrometer. Subsequently, a nebulizer converted the sample into tiny droplets in an argon aerosol, which then entered the ICP. After passing through a focusing region, the ions proceeded into the dynamic reaction cell (DRC) and eventually through the quadrupole mass filter. The detector permitted the selective counting of ions in rapid succession to detect and identify isotopes of individual elements. Detailed experimental protocols and techniques have been recorded on the NHANES Experimental Protocols webpage. According to the NHANES standard, metal concentrations that fell below the detection limit were replaced by the detection limit divided by the square root of two. In addition, our analysis corrected the metal concentrations in urine by accounting for urinary creatinine, and the results were expressed as μg/g creatinine.

### Covariates

We collected these covariates below by summarizing previous studies: age (20–59 years old, ≥ 60 years old), gender (male, female), race/ethnicity (Mexican American, other Hispanic, non-Hispanic white, non-Hispanic black, other race/multiracial), education level (under high school, high school or equivalent, above high school), marital status (married/cohabiting, widowed/divorced/separated, never married), family poverty income ratio (PIR) (≤ 1.30, 1.31–3.50, > 3.50), physical activity (none, moderate, vigorous), body mass index (BMI) (< 25, 25–29.99, ≥ 30 kg/m^2^), waist circumference, serum cotinine (≤ 0.011, > 0.011 ng/mL), alcohol use (never, yes), and NHANES cycles. Medical examinations were carried out at mobile examination centers, and blood and urine samples were taken. Moderate intensity physical activities refer to activities that cause a slight increase in breathing or heart rate, such as brisk walking or carrying light loads continuously. Vigorous physical activity refers to activities that involve a significant increase in breathing or heart rate, such as carrying or lifting heavy loads, digging, or construction work. Cotinine is the main metabolite of nicotine in the body and can serve as a biomarker of active smoking and tobacco exposure from secondhand smoke [28].

### Statistical Analysis

The baseline characteristics of the participants were assessed by SUI status using Chi-square tests and *t* tests. Categorical variables were presented as *n* (%) and continuous variables as mean ± standard deviation (SD). Due to the rightward deviation of heavy metal concentrations in humans, in order to approximate the normal distribution of heavy metal concentrations in blood and urine, they were Ln-transformed and categorized into four quartiles, denoted as Q1, Q2, Q3, and Q4. Spearman’s correlation analysis was used to determine the correlation among the Ln-transformed metals. A correlation coefficient of 0.00–0.09 indicates negligible correlation, 0.10–0.39 indicates weak correlation, 0.40–0.69 indicates moderate correlation, 0.70–0.89 indicates strong correlation, and 0.90–1.00 indicates very strong correlation [29]. Additionally, we conducted a stratified analysis based on age and gender, dividing the participants into young and middle-aged (20–59 years old) and elderly group (≥ 60 years old), male and female groups.

Multivariate logistic regression was employed to explore the relationship between single metals and SUI. The reference group was defined as the first quartile (Q1), and the results were presented as odds ratios (OR) along with their corresponding 95% confidence intervals (OR, (95%CI)). The model corrected for all potential confounders, including age, gender, race/ethnicity, education level, marital status, family PIR, physical activity, BMI, waist circumference, serum cotinine, alcohol use, and NHANES cycles. In addition, to control for the influence of other metals, we also constructed separate multivariate logistic regression models incorporating 10 blood and 3 urinary metals, thereby improving the robustness of the results.

Typically, people come into contact with multiple metals simultaneously [16]. In this study, we used WQS regression to analyze whether metal mixtures in blood or urine were associated with SUI. If relevant, it was further analyzed which metals played a key role. In WQS regression analysis, all data were randomly grouped into training and validation sets. The bootstrap was run 1000 times in the training set to acquire the weights for each metal, and the mixtures were checked for significance in the validation set. The WQS index (ranged from 0 to 1) was composed of the weighted sum of individual metal concentrations, calculated based on experience by the R software package (“gWQS”). It represents the mixed exposure level of 3 blood metals or 10 urinary metals, with the final result being interpreted as a simultaneous effect of a one-quartile increase in the mixture of metals on SUI.

In addition, the qgcomp model was further introduced in this study to overcome the main limitations of the WQS model in the direction of association. This model was completed by the R package “qgcomp”, which combined the reasoning simplicity of WQS regression with the flexibility of g-computation without the assumptions of homogeneity of directions and the linearity and additivity of the exposures and the ability to compute positive and negative weights for each variable in the mixture.

BKMR was employed to examine the joint effects and potential interactions between blood and urinary metals and SUI risk. The BKMR model is a flexible and powerful emerging method of statistical analysis that does not require setting up parametric expressions and allows for the presence of non-linear effects and interactions. The method generated kernel functions from the mixture variables that were put into the model and then generated relationship curves (dose–response curves) between the mixture components and the SUI variables using Bayesian sampling and analysis methods. The importance of each single metal in the heavy metal mixture can be derived by calculating the posterior inclusion probability (PIP) [30]. Estimates from the BKMR model were computed after 20,000 iterations employing the R software package (“bkmr”).

RCS analysis is widely recognized as an efficient statistical tool for describing the dose–response relationship between sustained exposure to a substance and specific outcome indicators [31]. We used this statistical method to assess the relationship between each metal exposure and SUI, with three knots set at the 5th, 50th, and 95th percentiles of metal exposure levels. The corresponding metal concentration based on an OR value of 1 in the sample was used as the reference concentration, which was then utilized to calculate the different OR values for the other metal concentrations. The R software package “rms” was employed to identify significant correlations between blood and urinary metal concentrations and ORs.

## Results

### Study Population Characteristics

Of the 10,622 participants in NHANES 2003–2018, 2455 (23.11%) were diagnosed with SUI, 2331 (21.94%) with UUI, and 1064 (10.02%) with MUI. Among SUI, 1473 were young and middle-aged, 982 were elderly, 2201 were female, and 254 were male. Table 1 lists the basic characteristics of participants with SUI in the United States. In general, age, gender, race/ethnicity, marital status, physical activity, BMI, waist circumference, serum cotinine, and alcohol use showed statistically significant differences between participants with and without SUI.

**Table 1:** Characteristics of participants in the NHANES 2003–2018

Characteristics	Total *N* = 10622	With SUI *n* = 2455 (23.1%)	Without SUI *n* = 8167 (76.9%)	*p* value
Age, *N* (%)				** < 0.001**
20–59	7034 (66.2)	1473 (60.0)	5561(68.1)	
≥ 60	3588 (33.8)	982 (40.0)	2606 (31.9)	
Gender, *N* (%)				** < 0.001**
Male	5332 (50.2)	254 (10.3)	5078 (62.2)	
Female	5290 (49.8)	2201 (89.7)	3089 (37.8)	
Race/ethnicity, *N* (%)				** < 0.001**
Mexican American	1679 (15.8)	404 (16.5)	1275 (15.6)	
Other Hispanic	899 (8.5)	206 (8.4)	693 (8.5)	
Non-Hispanic white	4921 (46.3)	1281 (52.2)	3640 (44.6)	
Non-Hispanic black	2147 (20.2)	381 (15.5)	1766 (21.6)	
Other race/multiracial	976 (9.2)	183 (7.4)	793 (9.7)	
Educational level, *N* (%)				**0.55**
Under high school	2535 (23.9)	596 (24.3)	1939 (23.7)	
High school or equivalent	2500 (23.5)	558 (22.7)	1942 (23.8)	
Above high school	5587 (52.6)	1301 (53.0)	4286 (52.5)	
Marital status, *N* (%)				** < 0.001**
Married/cohabiting	6465 (60.9)	1448 (59.0)	5017 (61.4)	
Widowed/divorced/separated	2331 (21.9)	743 (30.2)	1588 (19.5)	
Never married	1826 (17.2)	264 (10.8)	1562 (19.1)	
Family PIR, *N* (%)				**0.45**
≤ 1.30	3162 (29.8)	754 (30.7)	2408 (29.5)	
1.31–3.50	4136 (38.9)	934 (38.1)	3202 (39.2)	
> 3.50	3324 (31.3)	767 (31.2)	2557 (31.3)	
Physical activity, *N* (%)				** < 0.001**
None	5625 (53.0)	1374 (56.0)	4251 (52.1)	
Moderate	2605 (24.5)	691 (28.1)	1914 (23.4)	
Vigorous	2392 (22.5)	390 (15.9)	2002 (24.5)	
BMI (kg/m^2^), *N* (%)				** < 0.001**
< 25	3038 (28.6)	544 (22.2)	2494 (30.5)	
25–29.99	3614 (34.0)	725 (29.5)	2889 (35.4)	
≥ 30	3970 (37.4)	1186 (48.3)	2784 (34.1)	
WC (cm), (mean ± SD)	99.49 (16.17)	101.85 (16.62)	98.78 (15.96)	** < 0.001**
Serum cotinine (ng/mL), N (%)				** < 0.001**
≤ 0.011	2765 (26.0)	744 (30.3)	2021 (24.7)	
> 0.011	7857 (74.0)	1711 (69.7)	6146 (75.3)	
Alcohol use, *N* (%)	3182 (30.0)	570 (23.2)	2612 (32.0)	** < 0.001**

Categorical variables were presented as *N* (%). *BMI*, Body mass index; *N*, numbers of subject; *NHANES*, National Health and Nutrition Examination Survey; *PIR*, poverty income ratio; *SD*, standard deviation; *SUI*, stress urinary incontinence; *WC*, waist circumference

### Heavy Metal Concentrations and Correlations

Table S1 presents the distributions of metal concentration in both blood and urine, encompassing the detection rate, median, minimum, mean, and maximum values. Except for blood Cd (72.19%), the detection rates of all other metals were more than 75%. Urine samples from nearly all participants showed detectable levels of Cs, Mo, and TI, with Mo having the highest concentrations. Compared with participants without SUI, SUI patients had significantly higher concentrations of blood Cd, as well as urinary Ba, Cd, Co, Cs, Mo, Pb, and TI (*p* values < 0.05). After the Ln transformation, the correlation between the metals was assessed using Spearman’s rank correlation coefficient (Fig. S2). In blood, Pb demonstrated a correlation coefficient of 0.34 with Cd and 0.12 with Hg, respectively. In urine, there were moderate correlations for several metals, including Cs with Tl (*r* = 0.59), Ba with Co (*r* = 0.44), and Cd with Pb (*r* = 0.42). The other correlations were below 0.4 and relatively weak.

### Logistic Regression to Analyze the Association Between Single Metal and SUI Risk

After correcting for all 12 covariates, the relationship between single metal and SUI risk was assessed using multivariate logistic regression, and the results were presented in Table 2. The highest tertiles (Q4) of blood Cd, Pb, and Hg significantly increased the SUI risk compared with Q1 (Cd: OR: 1.38, 95%CI: 1.18–1.62; Pb: OR: 1.44, 95%CI: 1.21–1.72; Hg: OR: 1.30, 95%CI: 1.11–1.53). Consistent with this, the odds of SUI increased by 19%, 23%, and 9% for each increasing unit of Ln-Cd, Ln-Pb, and Ln-Hg, respectively (*p* values < 0.05). In urinary metals, Q4 of Cd and Pb significantly increased the SUI risk compared with Q1 (Cd: OR: 1.77, 95%CI: 1.49–2.10; Pb: OR: 1.50, 95%CI: 1.27–1.79). The odds of SUI increased by 26% and 16% for each increasing unit of Ln-Cd and Ln-Pb in urine, respectively. In the subgroup analyses according to age and gender, the 20–59-year-old group and the female group showed the above consistent results. In addition, the 20–59-year-old group had an increased risk of SUI in Q4 (compared with Q1) of urinary Cs (OR: 1.35, 95%CI: 1.10–1.65), and this association was also present between ln-transformed metal concentration and the risk of SUI. In the male subgroup, there was an increased risk of SUI in Q4 (versus Q1) for blood Pb (OR: 1.88, 95%CI: 1.09–3.24) and urinary Pb (OR: 1.92, 95%CI: 1.22–3.01). There was no statistically significant association between other metals and the SUI.

**Table 2:** Associations of single blood and urinary metals with SUI risk in the NHANES 2003–2018

Metals	Q1	Q2		Q3		Q4		Continuous	
		OR (95% CI)	*P* value	OR (95% CI)	*P* value	OR (95% CI)	*P* value	OR (95% CI)	*P* value
Blood (μg/L)									
Cd									
Overall	Ref	1.20 (1.02–1.40)	**0.024**	1.15 (0.99–1.34)	0.069	1.38 (1.18–1.62)	** < 0.001**	1.19 (1.11–1.28)	** < 0.001**
Age (20–59)		1.23 (1.01–1.49)	**0.037**	1.16 (0.96–1.41)	0.123	1.60 (1.31–1.96)	** < 0.001**	1.28 (1.17–1.39)	** < 0.001**
Age ≥ 60		1.00 (0.75–1.32)	0.997	0.96 (0.74–1.24)	0.754	0.95 (0.72–1.26)	0.722	0.99 (0.87–1.12)	0.855
Male		0.98 (0.67–1.44)	0.938	0.91 (0.62–1.33)	0.612	1.25 (0.85–1.82)	0.255	1.22 (1.02–1.45)	**0.026**
Female		1.22 (1.03–1.45)	**0.025**	1.17 (0.99–1.38)	0.067	1.37 (1.14–1.63)	** < 0.001**	1.17 (1.08–1.27)	** < 0.001**
Pb									
Overall	Ref	1.25 (1.08–1.45)	**0.002**	1.37 (1.17–1.60)	** < 0.001**	1.44 (1.21–1.72)	** < 0.001**	1.23 (1.12–1.35)	** < 0.001**
Age (20–59)		1.38 (1.17–1.64)	** < 0.001**	1.62 (1.34–1.96)	** < 0.001**	1.68 (1.33–2.13)	** < 0.001**	1.38 (1.23–1.54)	** < 0.001**
Age ≥ 60		0.84 (0.61–1.16)	0.285	0.85 (0.62–1.17)	0.325	0.90 (0.65–1.25)	0.516	0.99 (0.84–1.16)	0.891
Male		1.40 (0.81–2.43)	0.226	1.65 (0.96–2.81)	0.068	1.88 (1.09–3.24)	**0.023**	1.21 (0.97–1.52)	0.092
Female		1.30 (1.11–1.52)	** < 0.001**	1.44 (1.22–1.71)	** < 0.001**	1.40 (1.15–1.70)	** < 0.001**	1.25 (1.13–1.38)	** < 0.001**
Hg									
Overall	Ref	1.38 (1.19–1.60)	** < 0.001**	1.28 (1.10–1.49)	**0.002**	1.30 (1.11–1.53)	** < 0.001**	1.09 (1.02–1.15)	**0.005**
Age (20–59)		1.53 (1.27–1.85)	** < 0.001**	1.40 (1.15–1.70)	** < 0.001**	1.45 (1.18–1.79)	** < 0.001**	1.14 (1.06–1.23)	** < 0.001**
Age ≥ 60		1.13 (0.89–1.44)	0.315	1.08 (0.85–1.39)	0.526	1.08 (0.84–1.39)	0.561	1.00 (0.91–1.10)	0.975
Male		1.30 (0.88–1.90)	0.184	1.21 (0.82–1.81)	0.341	1.36 (0.91–2.02)	0.134	1.05 (0.91–1.21)	0.480
Female		1.39 (1.19–1.64)	** < 0.001**	1.28 (1.09–1.51)	**0.003**	1.31 (1.10–1.56)	**0.003**	1.10 (1.03–1.17)	**0.005**
Urine (μg/g creatinine)									
Ba									
Overall	Ref	0.96 (0.82–1.12)	0.572	0.96 (0.82–1.12)	0.582	1.03 (0.88–1.20)	0.725	1.01 (0.95–1.07)	0.731
Age (20–59)		0.88 (0.72–1.08)	0.215	0.91 (0.74–1.11)	0.338	0.97 (0.79–1.18)	0.764	0.99 (0.92–1.07)	0.785
Age ≥ 60		1.10 (0.86–1.41)	0.437	1.06 (0.83–1.35)	0.662	1.15 (0.90–1.46)	0.260	1.05 (0.96–1.14)	0.279
Male		1.15 (0.81–1.64)	0.439	1.11 (0.76–1.62)	0.601	1.34 (0.93–1.95)	0.121	1.12 (0.98–1.29)	0.094
Female		0.93 (0.78–1.10)	0.399	0.95 (0.80–1.12)	0.527	0.99 (0.83–1.17)	0.885	1.00 (0.94–1.06)	0.940
Cd									
Overall	Ref	1.31 (1.11–1.55)	**0.002**	1.59 (1.35–1.88)	** < 0.001**	1.77 (1.49–2.10)	** < 0.001**	1.26 (1.18–1.35)	** < 0.001**
Age (20–59)		1.28 (1.06–1.55)	**0.011**	1.60 (1.32–1.95)	** < 0.001**	2.08 (1.69–2.56)	** < 0.001**	1.37 (1.25–1.49)	** < 0.001**
Age ≥ 60		0.95 (0.64–1.40)	0.786	1.01 (0.70–1.46)	0.949	0.99 (0.69–1.43)	0.974	1.02 (0.90–1.15)	0.788
Male		1.26 (0.85–1.88)	0.247	1.03 (0.67–1.58)	0.887	1.47 (0.96–2.27)	0.078	1.20 (1.00–1.44)	0.050
Female		1.27 (1.05–1.53)	**0.013**	1.63 (1.36–1.97)	** < 0.001**	1.76 (1.46–2.13)	** < 0.001**	1.25 (1.16–1.35)	** < 0.001**
Co									
Overall	Ref	1.07 (0.90–1.27)	0.449	1.13 (0.95–1.33)	0.157	1.13 (0.96–1.34)	0.147	1.04 (0.96–1.13)	0.294
Age (20–59)		1.17 (0.93–1.46)	0.189	1.17 (0.94–1.46)	0.155	1.06 (0.85–1.32)	0.589	0.98 (0.89–1.09)	0.768
Age ≥ 60		0.89 (0.68–1.16)	0.390	0.96 (0.74–1.24)	0.739	1.11 (0.86–1.45)	0.419	1.06 (0.94–1.20)	0.353
Male		1.22 (0.87–1.72)	0.253	1.28 (0.88–1.85)	0.200	1.37 (0.90–2.07)	0.143	1.08 (0.89–1.32)	0.423
Female		1.01 (0.83–1.24)	0.909	1.05 (0.86–1.27)	0.649	1.02 (0.85–1.24)	0.807	1.01 (0.93–1.10)	0.831
Cs									
Overall	Ref	0.92 (0.78–1.08)	0.305	1.11 (0.95–1.31)	0.194	1.16 (0.99–1.37)	0.071	1.22 (1.09–1.37)	** < 0.001**
Age (20–59)		0.81 (0.66–1.00)	**0.047**	1.10 (0.90–1.34)	0.361	1.35 (1.10–1.65)	**0.004**	1.45 (1.25–1.68)	** < 0.001**
Age ≥ 60		1.06 (0.79–1.41)	0.701	1.04 (0.79–1.38)	0.775	0.88 (0.67–1.17)	0.380	0.94 (0.79–1.12)	0.497
Male		0.97 (0.68–1.39)	0.881	1.12 (0.77–1.62)	0.561	0.93 (0.61–1.41)	0.729	1.08 (0.81–1.43)	0.594
Female		0.89 (0.74–1.07)	0.212	1.10 (0.92–1.32)	0.292	1.18 (0.99–1.42)	0.069	1.25 (1.11–1.41)	** < 0.001**
Mo									
Overall	Ref	0.97 (0.83–1.13)	0.724	0.96 (0.83–1.12)	0.632	0.95 (0.82–1.11)	0.540	0.96 (0.88–1.04)	0.344
Age (20–59)		1.03 (0.85–1.25)	0.760	1.01 (0.84–1.23)	0.899	0.92 (0.75–1.12)	0.382	0.95 (0.85–1.06)	0.345
Age ≥ 60		0.90 (0.70–1.16)	0.424	0.91 (0.70–1.17)	0.440	1.02 (0.80–1.30)	0.865	0.99 (0.87–1.13)	0.922
Male		0.83 (0.59–1.19)	0.317	0.93 (0.65–1.33)	0.678	0.88 (0.60–1.28)	0.493	0.87 (0.71–1.06)	0.171
Female		1.01 (0.85–1.19)	0.940	0.97 (0.82–1.15)	0.712	0.96 (0.81–1.14)	0.655	0.97 (0.89–1.07)	0.579
Pb									
Overall	Ref	1.43 (1.22–1.67)	** < 0.001**	1.35 (1.15–1.59)	** < 0.001**	1.50 (1.27–1.79)	** < 0.001**	1.16 (1.07–1.26)	** < 0.001**
Age (20–59)		1.45 (1.20–1.74)	** < 0.001**	1.46 (1.20–1.78)	** < 0.001**	1.65 (1.33–2.04)	** < 0.001**	1.27 (1.14–1.40)	** < 0.001**
Age ≥ 60		1.23 (0.89–1.70)	0.210	1.03 (0.75–1.42)	0.842	1.15 (0.83–1.60)	0.390	0.97 (0.85–1.11)	0.668
Male		1.43 (0.94–2.19)	0.096	1.06 (0.67–1.67)	0.804	1.92 (1.22–3.01)	**0.004**	1.28 (1.05–1.56)	**0.015**
Female		1.43 (1.20–1.69)	** < 0.001**	1.42 (1.19–1.69)	** < 0.001**	1.43 (1.19–1.73)	** < 0.001**	1.14 (1.04–1.24)	**0.005**
Sb									
Overall	Ref	1.08 (0.92–1.26)	0.334	1.03 (0.88–1.20)	0.686	1.07 (0.92–1.25)	0.389	1.03 (0.95–1.12)	0.480
Age (20–59)		1.09 (0.90–1.32)	0.391	1.01 (0.83–1.22)	0.955	1.05 (0.86–1.28)	0.651	1.00 (0.90–1.11)	0.947
Age ≥ 60		1.07 (0.83–1.38)	0.589	1.08 (0.84–1.39)	0.535	1.14 (0.89–1.47)	0.303	1.10 (0.97–1.25)	0.150
Male		1.25 (0.87–1.78)	0.226	1.16 (0.80–1.69)	0.428	1.18 (0.79–1.75)	0.423	1.09 (0.90–1.33)	0.380
Female		1.04 (0.88–1.24)	0.626	1.01 (0.85–1.20)	0.882	1.07 (0.90–1.27)	0.461	1.03 (0.94–1.13)	0.485
TI									
Overall	Ref	0.96 (0.81–1.12)	0.576	0.98 (0.83–1.14)	0.755	0.99 (0.85–1.16)	0.925	1.04 (0.94–1.14)	0.490
Age (20–59)		1.03 (0.83–1.27)	0.820	1.03 (0.84–1.27)	0.782	1.07 (0.87–1.32)	0.516	1.09 (0.96–1.25)	0.188
Age ≥ 60		0.91 (0.71–1.16)	0.433	0.93 (0.73–1.19)	0.571	0.91 (0.71–1.16)	0.435	0.97 (0.83–1.13)	0.692
Male		1.15 (0.83–1.60)	0.402	1.10 (0.77–1.57)	0.587	0.92 (0.60–1.40)	0.694	1.02 (0.79–1.31)	0.872
Female		0.94 (0.78–1.13)	0.512	0.97 (0.81–1.15)	0.696	1.01 (0.85–1.19)	0.951	1.05 (0.95–1.18)	0.331
Tu									
Overall	Ref	0.96 (0.83–1.12)	0.640	0.92 (0.80–1.07)	0.299	0.86 (0.74–1.00)	0.053	0.96 (0.90–1.02)	0.187
Age (20–59)		1.08 (0.89–1.31)	0.453	0.88 (0.73–1.07)	0.206	0.86 (0.71–1.05)	0.132	0.95 (0.88–1.03)	0.204
Age ≥ 60		0.83 (0.65–1.06)	0.135	1.02 (0.80–1.29)	0.897	0.88 (0.69–1.13)	0.321	0.98 (0.89–1.09)	0.750
Male		0.73 (0.50–1.05)	0.088	1.11 (0.79–1.57)	0.549	1.03 (0.71–1.49)	0.891	1.04 (0.89–1.21)	0.619
Female		1.01 (0.86–1.20)	0.868	0.89 (0.75–1.05)	0.170	0.84 (0.71–0.99)	**0.033**	0.94 (0.88–1.01)	0.098
As									
Overall	Ref	1.06 (0.91–1.23)	0.474	1.07 (0.92–1.25)	0.362	1.05 (0.90–1.23)	0.531	1.01 (0.96–1.07)	0.642
Age (20–59)		1.13 (0.93–1.37)	0.207	1.15 (0.94–1.39)	0.169	1.09 (0.89–1.33)	0.405	1.02 (0.95–1.09)	0.630
Age ≥ 60		0.93 (0.72–1.20)	0.574	0.93 (0.72–1.21)	0.602	0.96 (0.74–1.23)	0.739	1.00 (0.92–1.09)	0.938
Male		1.19 (0.83–1.71)	0.337	0.98 (0.66–1.44)	0.901	1.18 (0.81–1.73)	0.388	1.06 (0.92–1.21)	0.428
Female		1.03 (0.87–1.21)	0.768	1.08 (0.91–1.28)	0.355	1.03 (0.87–1.22)	0.738	1.01 (0.95–1.07)	0.767

Models were adjusted for age, gender, race/ethnicity, education levels, marital status, poverty income ratio, physical activity, body mass index, waist circumference, serum cotinine, alcohol use, and NHANES cycles. Continuous, Ln-transformed concentration of metal; *CI* confidence interval; *OR* odds ratio; *Q* quartile; *Ref* reference. Bold: *p* < 0.05

To adjust for the confounding effects of other metals, we constructed separate multivariate logistic regression models which included all blood and urine metals. Similar results were observed in this sensitivity analysis, with the Q4 of blood Cd, Pb, Hg, and urinary Cd, Pb significantly increasing the risk of developing SUI compared with Q1 (blood Cd: OR: 1.29, 95% CI: 1.09–1.52; Pb: OR: 1.34, 95% CI: 1.12–1.61; Hg: OR: 1.27, 95% CI: 1.08–1.49; urinary Cd: OR: 1.64, 95% CI: 1.37–1.97; Pb: OR: 1.34, 95% CI: 1.11–1.62) (Table S2).

### WQS and qgcomp Models to Assess the Association Between Metals Co-exposure and SUI Risk

**Fig. 1:**
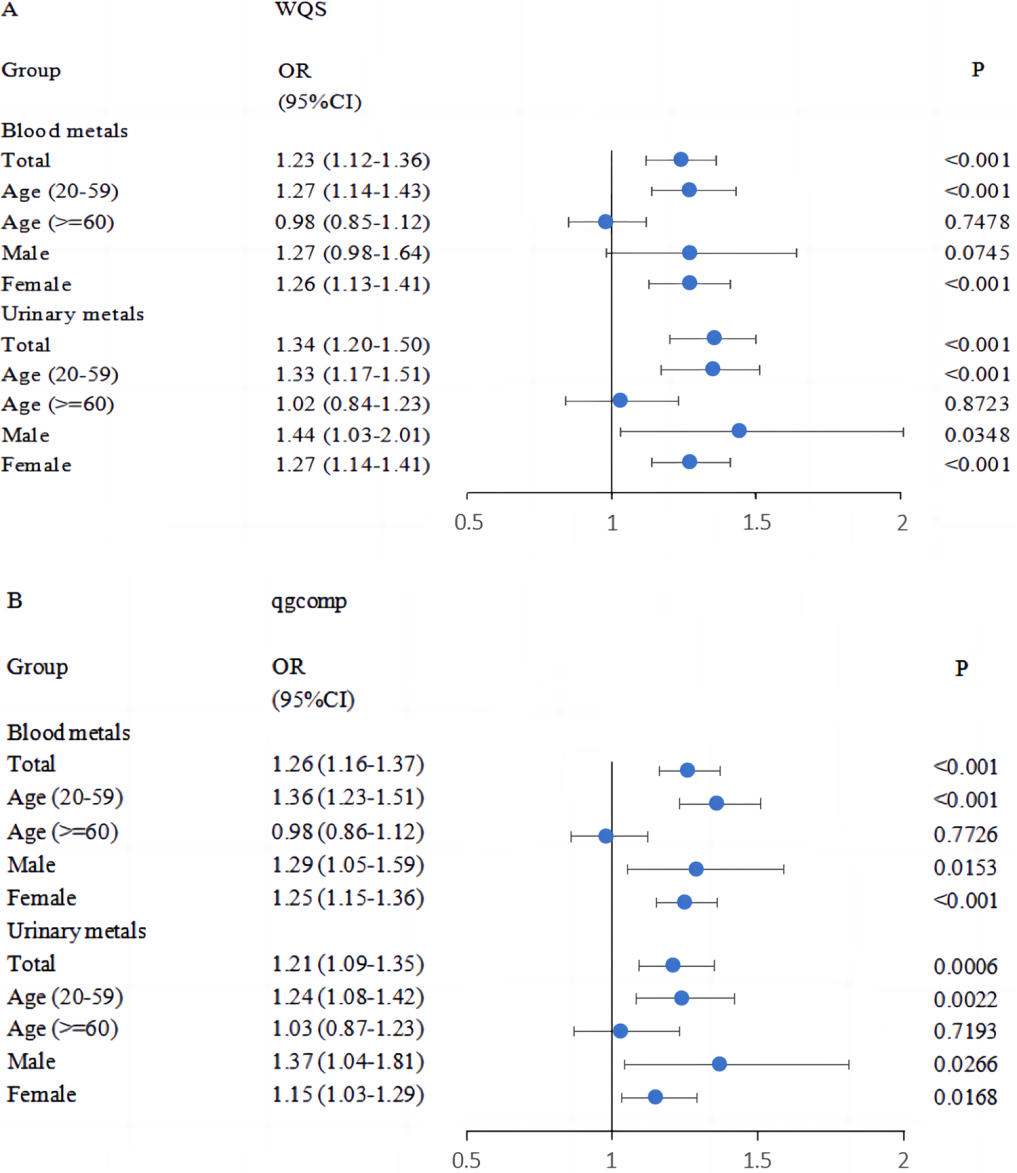
Odds ratios (95%CI) of SUI associated with co-exposure to blood and urinary metal mixtures by WQS (**A**) and qgcomp (**B**) analyses. Models were adjusted for age, gender, race/ethnicity, education levels, marital status, poverty income ratio, physical activity, body mass index, waist circumference, serum cotinine, alcohol use, and NHANES cycles

The WQS model, after adjusting for all 12 covariates, showed that the WQS index for three blood metals and ten urinary metals were positively associated with the risk of SUI in the total (blood metals OR: 1.23, 95% CI: 1.12–1.36; urinary metals OR: 1.34, 95% CI: 1.20–1.50), 20–59 years old group (blood metals OR: 1.27, 95% CI: 1.14–1.43; urinary metals OR: 1.33, 95% CI: 1.17–1.51), and female group (blood metals OR: 1.26, 95% CI: 1.13–1.41; urinary metals OR: 1.27, 95%CI: 1.14–1.41) (Fig. 1A). In the male group, only the urinary mixture was statistically significant (urinary metals OR: 1.44, 95% CI: 1.03–2.01). This suggests that co-exposure to blood metals or urinary metals had a stimulative effect on SUI. Inversely, it was not statistically significant in the elderly group (blood metals OR: 0.98, 95% CI: 0.85–1.12; urinary metals OR: 1.02, 95% CI: 0.84–1.23). Figure S3 shows the estimated weight of each metal for all WQS index. In the total population, 20–59-year-old group and female group, the metal with the highest weight in the blood metal mixture was Pb, and in the urinary, metal mixture was Cd.

In sensitivity analysis, the qgcomp regression model was used to estimate exposure weights in both the positive and negative directions. The results showed that co-exposure to blood and urinary metals remained significantly correlated with SUI in the total (blood metals OR: 1.26, 95% CI: 1.16–1.37; urinary metals OR: 1.21, 95% CI: 1.09–1.35), 20–59 years old (blood metals OR: 1.36, 95% CI: 1.23–1.51; urinary metals OR: 1.24, 95% CI: 1.08–1.42), female (blood metals OR: 1.25, 95% CI: 1.15–1.36; urinary metals OR: 1.15, 95% CI: 1.03–1.29) and male groups (blood metals OR: 1.29, 95% CI: 1.05–1.59; urinary metals OR: 1.37, 95% CI: 1.04–1.81), but not with the elderly group (blood metals OR: 0.98, 95% CI: 0.86–1.12; urinary metals OR: 1.03, 95% CI: 0.87–1.23) (Fig. 1B). The positive and negative weights for each metal were presented in Fig. S4, with Pb being the most dominant positive driver in the blood metal mixture and Cd in the urinary metal mixture.

### BKMR Model to Assess the Association Between Metals Co-exposure and SUI Risk

**Fig. 2:**
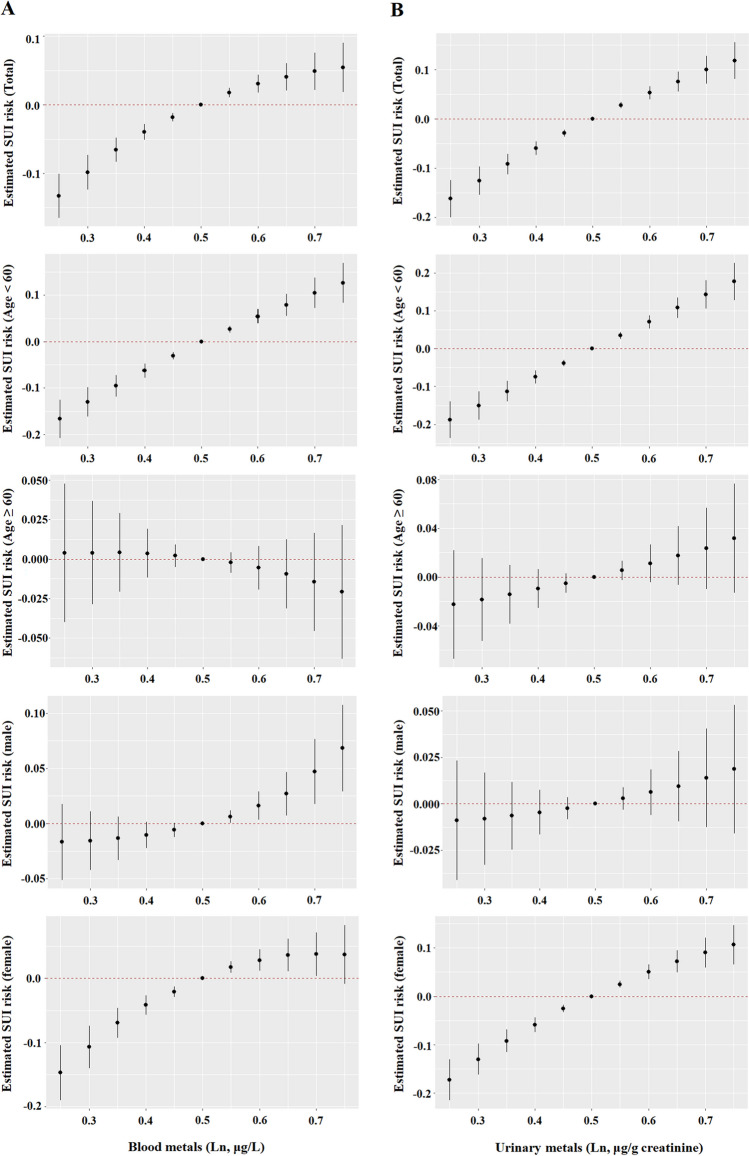
The joint effects of blood (**A**) and urinary (**B**) metal mixtures on SUI risk were estimated by BKMR models in total population and subgroups, when all the metals at particular percentiles were compared to all the metals at their 50th percentile. Models were adjusted for age, gender, race/ethnicity, education levels, marital status, Poverty Income Ratio, physical activity, body mass index, waist circumference, serum cotinine, alcohol use, and NHANES cycles

**Fig. 3:**
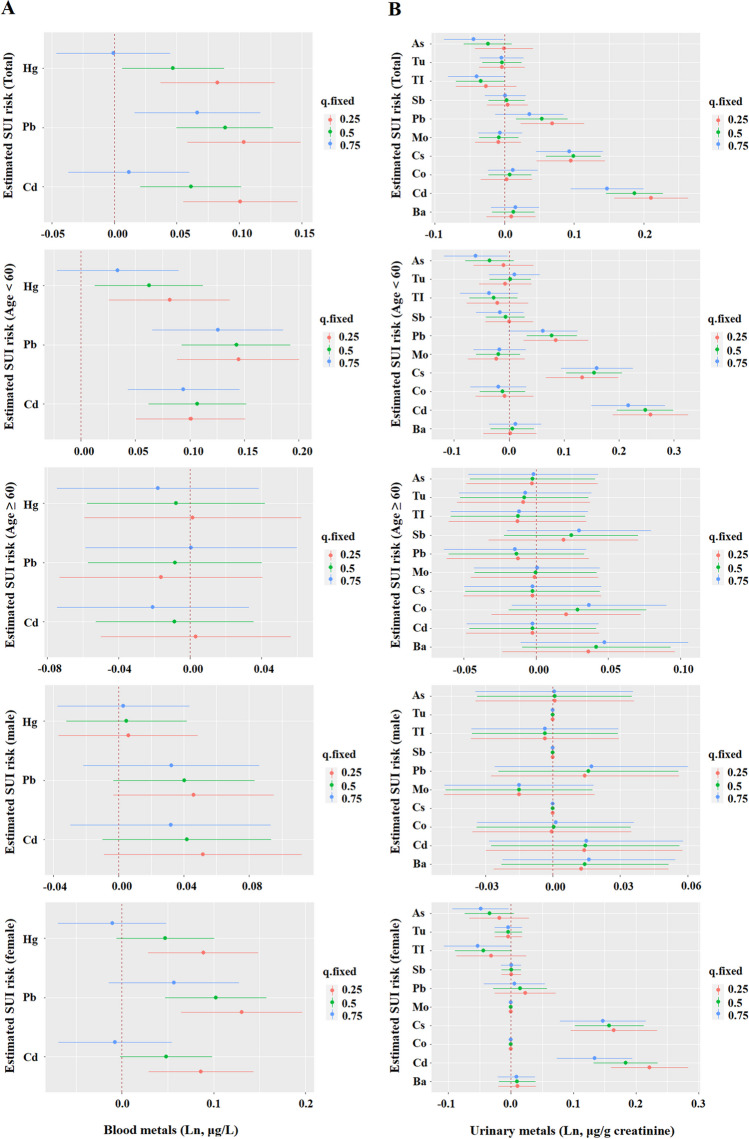
Associations of single blood (**A**) and urinary (**B**) metals with SUI risk were estimated by BKMR models in total population and subgroups, when other all metals were held at their corresponding 25th (red), 50th (green) or 75th (blue) percentile, respectively. Models were adjusted for age, gender, race/ethnicity, education levels, marital status, poverty income ratio, physical activity, body mass index, waist circumference, serum cotinine, alcohol use, and NHANES cycles

Figure 2 shows the overall correlation between metal mixtures and increased risk of SUI. The overall exposure–response function in the total population group and the 20–59-year-old group showed that blood metal mixtures were significantly and positively associated with SUI risk, with PIP values greater than 0.9 for Cd, Pb, and Hg (Fig. 2A and Table S3). In addition, we found that Pb has a significantly positive effect on SUI risk when the concentrations of all remaining metals were fixed at the 25th, 50th, and 75th percentiles, while Cd and Hg had a significantly positive effect when the concentrations of all remaining metals were fixed at the 25th and 50th percentiles. Among participants aged 20–59 years, Cd and Pb had a significantly positive effect on SUI risk when all remaining metals were fixed at the 25th, 50th, and 75th percentiles, and Hg had a positive effect when metals were fixed at the 25th and 50th percentiles (Fig. 3A).

As for urinary metal mixtures, when their concentration reached or exceeded the 55th percentile, there was a significant combined toxic effect on SUI compared with the median in the total population, 20–59 years old group, and female group (Fig. 2B). We further found that when all remaining metal concentrations were fixed at the 25th, 50th, and 75th percentiles, only Cd and Cs had a significantly positive effect on increasing the risk of SUI, suggesting that Cd and Cs may drive the overall function (Fig. 3B).

Considering that there may be a certain degree of correlation between some metals, we further analyzed the interactions between three blood metals and 10 urinary metals respectively. The results indicate that there may be potential interactions among several metals in blood and urine (e.g., blood Cd and Hg; urinary Cd and As, TI) (Fig. S6).

### RCS Regression to Assess the Dose–Response Relationship Between Metals and SUI Risk

The RCS regression was used to analyze the dose–response relationships between single metals and SUI risk, as detailed in Fig. S7–S12. Among the blood metals, there was a linear relationship between Cd concentration and SUI risk (*p*nonlinear = 0.1544). The risk of SUI increases with the rise of blood Cd levels (*p*overall < 0.0001). A similar dose–response relationship was also found in the 20–59-year-old group (*p*nonlinear = 0.2050, *p*overall < 0.0001) and female group (*p*nonlinear = 0.4461, *p*overall = 0.0003), but not in the elderly and male group. In urinary metals, a linear relationship was found between Cs concentration and SUI risk in the total (*p*nonlinear = 0.6409, *p*overall = 0.0017), 20–59-year-old group (*p*nonlinear = 0.0877, *p*overall < 0.0001), and female group (*p*nonlinear = 0.5742, *p*overall = 0.0015). Urinary Cd showed a linear relationship with SUI risk only in the 20–59-year-old group (*p*nonlinear = 0.5557, *p*overall < 0.0001).

## Discussion

Our study is the first to use multiple statistical methods to investigate the joint effects of metal mixtures in blood and urine on SUI risk in a big-sample data nationally representative. We observed significantly higher concentrations of Cd in blood and Ba, Cd, Co, Cs, Mo, Pb, and TI in urine among SUI participants compared to those without SUI. In the single-metal logistic regression analysis, Cd, Pb, and Hg in blood and Cd, Pb, and Cs in urine were identified as independent risk factors for SUI. In multiple logistic regression, the above metals remained significantly positively associated with SUI risk. Furthermore, the BKMR model and WQS regression consistently indicated a significant positive relationship between blood or urinary metal co-exposure and SUI risk. Notably, Pb and Cd in blood were considered the most substantial factors driving the overall effect, while Cd and Cs are regarded in urine. These findings were further confirmed by the qgcomp regression and RCS regression analysis.

Cd is a toxic heavy metal prevalent in the environment and widely distributed in media such as aquatic, industrial products, soil, plants, and food [32, 33]. Cd enters the human body through various pathways and accumulates mainly in the kidneys, pancreas, liver, bones, and central nervous system [34, 35], with nearly 50% of the accumulation in the kidneys [36]. Emerging evidence suggests that Cd accumulation in the kidneys can lead to kidney damage and subsequent kidney diseases, including nephritis, kidney stones, and chronic kidney disease [37, 38]. Consistent with these research findings, we found a positive correlation between Cd in blood and urine and the risk of SUI, which may be related to Cd-induced OS [39, 40]. OS plays a pivotal role in the development of SUI [41]. OS is a state of imbalance between oxidative and antioxidant effects in the body. In this state of imbalance, excess reactive oxygen species (ROS) can destroy cellular proteins, lipids, and DNA, leading to lethal cellular damage [42]. According to recent research findings, OS may cause detrusor overactivity and bladder overactivity, which are closely related to SUI [43]. Higher intake of nutrients such as calcium, zinc, magnesium, and selenium, as well as vitamins C, D, and E can contribute to resisting OS [44]. On the contrary, unhealthy lifestyles like smoking may accelerate OS-related cellular damage [45]. Moreover, smoking is an important source of Cd exposure in populations with nonoccupational exposure, as the majority of cigarettes contain approximately 1–2 μg of cadmium [37, 46]. Smoking excessively not only leads to a large accumulation of Cd in the kidneys, but also causes a variety of respiratory diseases such as lung cancer and chronic obstructive pulmonary disease (COPD). Consequently, smoking cessation is recommended as one of the lifestyle modifications for managing SUI [47]. However, we are exposed to Cd from a wide range of sources, and reducing passive exposure requires effective government measures and policies to control it [16].

In our study, Pb in blood and urine was also confirmed to be another toxic heavy metal contributing to the increased risk of SUI. Pb is one of the most serious environmental pollutants and a heavy metal element that seriously endangers human health. Due to its unique physical and chemical qualities, Pb is widely used in our daily lives and industries such as mining, storage batteries, cable protection sheaths, machine building, ceramics, light industry, and lead oxide [48, 49]. In addition, the informal practice of recycling metals from waste is widespread, especially in less developed countries, which has led to more exposure, illness, and even deaths [50]. It is estimated that adults could absorb 3–10% of water-soluble Pb at an oral dose, while the absorption rate may increase to 50% in pregnant women and children [51, 52]. The kidneys are the main organ for the accumulation of ingested Pb, which impedes glomerular development and causes irreversible nephrotoxicity when ingested over a long period [53]. In addition, Pb can induce mitochondrial dysfunction leading to increased levels of OS, and studies have demonstrated that Pb exposure can cause the mitochondrial permeability transition pore (MPTP) to open leading to apoptosis [54]. Also, Pb ions competitively inhibit the action of calcium ions in cells, disrupting cellular function [55]. The lead-induced increase in OS and disruption of calcium homeostasis may cause abnormal muscle contraction and chronic inflammatory response, which may be the mechanism of Pb exposure leading to the development of SUI [25].

Hg is also a nephrotoxic metal that is found ubiquitously in many environmental and certain occupational settings [56]. Humans are most commonly exposed to organic forms of Hg, such as methylmercury (CH3Hg +), through the consumption of contaminated food [37]. Once ingested, a portion of CH3Hg + is oxidized to form inorganic mercury (Hg2 +). Both forms of Hg have been shown to accumulate in the kidneys, leading to decreased glomerular filtration rate, tubular damage and necrosis, and destruction of renal function [57]. Like Pb, Hg causes mitochondrial damage and affects cellular functions [58]. In our results, we found a statistically significant association between blood Hg levels and the occurrence of SUI, contrary to the findings of Ni et al. [25].

Cs is a naturally occurring alkali metal in the environment. Once in the human body, it is distributed throughout the system, with particularly high concentrations in the kidneys, skeletal muscle, liver, and red blood cells [59]. Cs is mainly eliminated through the kidneys, and its excretion mechanism is similar to that of potassium [60]. To date, fewer studies have been conducted on the correlation between Cs exposure levels and SUI. In this study, we found for the first time that Cs exposure may increase the risk of SUI, and Cs to be one of the most positive factors influencing the overall effect of urinary metals. Although the exact mechanism of association between urinary Cs and SUI is unclear, some possible speculations exist as follows. There is a correlation between high concentrations of urinary Cs and elevated biomarkers of OS [61], and OS has been suggested to possibly play a significant role in detrusor overactivity and overactive bladder [62, 63]. In addition, a population-based repeated measures study found that Cs may disrupt the balance of inflammatory mediators and trigger inflammation [64], which is also a possible factor for Cs to increase the risk of SUI. Future studies still need to focus more on the relationship between Cs, including blood Cs, and SUI and further explore the potential mechanisms that increase the risk of SUI.

Although it has been confirmed that exposure to certain individual metals is associated with SUI risk [25], there is currently scarce information on the combined effects of metal mixtures. The impact of metals on diseases typically relies on their collaboration and interaction with one another. Epidemiological evidence indicates that co-exposure to multiple toxic heavy metals can cause OS, biomedical, and hematological changes, which can have various adverse effects on health [65]. Therefore, our study employed a variety of recently developed analytical approaches to reveal the common effects of multiple heavy metals on SUI. Among these, the WQS regression was specifically developed to estimate the impact of co-exposure by determining the weight of each metal within the metal mixture. Still, it is limited to simultaneously estimating joint effects in different directions, whereas the qgcomp model may lead to counteracting effects between exposures. Besides, the BKMR model was used to identify nonlinear and nonadditive effects of mixed exposures and interactions. In our study, the results of the three methods were in general agreement. Both blood and urinary metal mixtures contribute to the increased risk of SUI, with Pb and Cd playing a major role in the blood metal mixtures for the total effect, and Cd and Cs playing a key role in the urinary metal mixtures. Of interest, the BKMR model observed potential interactions between certain metals. Although the exact biological mechanisms are unknown, they may be related to OS activity and disruption of metal homeostasis [66]. In addition, RCS modeling was carried out to ensure the credibility of the findings. We found a positive linear relationship between blood Cd and urinary Cs and the risk of SUI.

Epidemiologic studies have shown differences in the etiopathogenesis, incidence, and clinical pattern of SUI among different age and gender groups. Thus, we further conducted stratified analyses by age and gender to elucidate the metal burden in SUI for each subgroup. Our results showed that more metals were significantly associated with SUI in the 20–59-year-old group and female group, compared to the elderly and male group. Moreover, the mixed exposure model showed consistent results that metal mixtures significantly increased the risk of SUI in both the 20–59-year-old group and female group. This may be because young and middle-aged individuals are more likely to be exposed to and affected by heavy metals in the environment than elderly people. Firstly, young and middle-aged people are usually at the peak of their careers and are likely to engage in more jobs and activities that involve exposure to heavy metals, such as industrial production, building construction, and electronics production [67]. At the same time, they have more socialization and outdoor activities, and may come into contact with contaminated soil and water bodies, increasing the likelihood of heavy metal exposure. In addition, young and middle-aged people have higher consumption power and are more inclined to consume foods such as seafood, which may also be at risk of heavy metal contamination. Therefore, young and middle-aged people need to pay more attention to exposure to heavy metals in the environment and the potential effects on SUI, and take preventive and management measures accordingly. Compared to men, women generally have higher concentrations of Cd in blood, urine, and kidneys [68]. One possible explanation is that Cd and iron share a common mechanism of uptake and there is transport competition at renal entry pathways [69]. Thus, inadequate iron stores and iron deficiency are prevalent in women globally, which may increase Cd absorption in the gastrointestinal tract and promote renal reabsorption of Cd, thereby augmenting the body and kidney Cd burden. In addition, limited data and animal studies suggest that women are more susceptible than men after exposure to toxic heavy metals [70]. However, gender-related disparities in metal exposure and its impact on health are highly overlooked areas of research that need to be given high priority in the future.

Our study has several significant strengths. First, the independent effects of blood and urinary heavy metal exposure on SUI risk were comprehensively assessed, filling a previous gap. Furthermore, we used five statistical methods in a larger population to explore the association between co-exposure to heavy metals and SUI risk from different perspectives and ultimately produced relatively consistent results. However, there are some unavoidable limitations to our study as well. First, cross-sectional studies are considered incapable of making causal inferences. Second, the diagnosis of SUI relied on self-reported questionnaires, which may be influenced by inherent biases. Third, due to data restrictions in the NHANES database, we are unable to determine the frequency and extent of heavy metal exposure among participants. Finally, our research did not explore the underlying pathological mechanisms between heavy metal exposure and the development of SUI.

## Conclusion

To summarize, we have discovered multiple metals that exhibit a statistically significant association to the risk of SUI. These metals include Cd, Pb, and Hg in the blood, as well as Cd, Pb, and Cs in the urine. Mixed exposure analyses consistently revealed that blood and urinary metal–mixed exposure increased the risk of SUI, with blood Pb and Cd, and urinary Cd and Cs being the main positive drivers, respectively. These associations were also observed in the 20–59 years old group and female group. However, despite the clinical and epidemiological significance of the results of this study, we must also recognize the inevitable limitations that exist. Several aspects deserve further exploration in future studies. First, more extensive prospective and experimental studies are needed to validate our findings and confirm the causal relationship between heavy metals exposure and SUI. Second, it is important to consider the influence of other potential environmental and lifestyle factors on SUI and explore possible interventions to reduce the risk of metal exposure and SUI. Finally, research on specific populations, such as children, pregnant women, and special occupations, needs to be strengthened to provide more targeted strategies for the prevention and treatment of SUI.

## Supplementary Information

Below is the link to the electronic supplementary material.

Supplementary file1 (DOCX 8.23 MB)

## Data Availability

Publicly available datasets were analyzed in this study. This data can be found here: National Health and Nutrition Examination a (http://www.cdc.gov/nchs/nhanes.htm).

## Abbreviations

NHANES: National Health and Nutrition Examination Survey
UI: Urinary incontinence
SUI: Stress urinary incontinence
UUI: Urge urinary incontinence
MUI: Mixed urinary incontinence
OS: Oxidative stress
LUTS: Lower urinary tract symptoms
WQS: Weighted quantile sum
Qgcomp: Quantile-based g-computation
BKMR: Bayesian kernel machine regression
RCS: Restricted cubic spline
Cd: Cadmium
Pb: Lead
Hg: Mercury
Ba: Barium
Co: Cobalt
Cs: Cesium
Mo: Molybdenum
Sb: Antimony
TI: Thallium
Tu: Tungsten
As: Arsenic
ICP-MS: Inductively coupled plasma mass spectrometry
DRC: Dynamic reaction cell
PIR: Poverty income ratio
BMI: Body mass index
SD: Standard deviation
OR: Odds ratios
CI: Confidence intervals
ROS: Reactive oxygen species
COPD: Chronic obstructive pulmonary disease
MPTP: Mitochondrial permeability transition pore
