# Supplementary material for: Associations of Blood and Urinary Heavy Metals with Stress Urinary Incontinence Risk Among Adults in NHANES, 2003–2018
Source: Biol Trace Elem Res. 2024 Jun 17;203(3):1327–41. doi: 10.1007/s12011-024-04264-8 (PMC11872759; doi:10.1007/s12011-024-04264-8)
Supplement: Supplementary file 1 — Supplementary file1 (DOCX 8.23 MB) [file 12011_2024_4264_MOESM1_ESM.docx]

***Supplementary materials***

**Table S1.** The distributions of 13 metal concentrations in blood and urine in the NHANES 2003-2018

| Metals | N | Detection  rates (%) | Total (n = 10622) | | | | With SUI (n = 2455) | | | | Without SUI (n = 8168) | | | |  |
| --- | --- | --- | --- | --- | --- | --- | --- | --- | --- | --- | --- | --- | --- | --- | --- |
|  |  |  | Median (IQR) | Min | Mean | Max | Median (IQR) | Min | Mean | Max | Median (IQR) | Min | Mean | Max | p |
| **Blood (μg/L)** | | | | | | | | | | | | | | | |
| Cd | 10622 | 72.19 | 0.32(0.39) | 0.07 | 0.50 | 8.67 | 0.36(0.40) | 0.07 | 0.57 | 8.67 | 0.31(0.37) | 0.07 | 0.49 | 8.18 | * |
| Pb | 10622 | 99.68 | 1.20(1.18) | 0.05 | 1.60 | 33.67 | 1.12(1.04) | 0.10 | 1.41 | 23.60 | 1.24(1.23) | 0.05 | 1.66 | 33.67 | * |
| Hg | 10622 | 79.45 | 0.85(1.21) | 0.10 | 1.51 | 63.64 | 0.84(1.09) | 0.10 | 1.39 | 30.70 | 0.85(1.26) | 0.10 | 1.55 | 63.64 | * |
| **Urine (μg/g creatinine)** | | | | | | | | | | | | | | | |
| Ba | 10622 | 98.59 | 1.21(1.57) | 0.02 | 1.96 | 124.7 | 1.40(1.85) | 0.04 | 2.29 | 97.77 | 1.16(1.49) | 0.02 | 1.86 | 124.7 | * |
| Cd | 10622 | 83.92 | 0.24(0.31) | 0.01 | 0.35 | 9.01 | 0.32(0.35) | 0.02 | 0.43 | 4.04 | 0.22(0.28) | 0.01 | 0.32 | 9.01 | * |
| Co | 10622 | 99.29 | 0.34(0.29) | 0.02 | 0.53 | 143.7 | 0.42(0.36) | 0.03 | 0.65 | 62.34 | 0.32(0.27) | 0.02 | 0.49 | 143.7 | * |
| Cs | 10622 | 99.99 | 4.25(2.79) | 0.04 | 5.10 | 581.2 | 4.89(3.13) | 0.64 | 6.12 | 581.2 | 4.09(2.63) | 0.04 | 4.79 | 164.7 | * |
| Mo | 10622 | 99.98 | 38.53(31.46) | 1.02 | 48.05 | 494.1 | 40.70(33.72) | 3.17 | 51.40 | 494.1 | 37.84(30.79) | 1.02 | 47.04 | 463.6 | * |
| Pb | 10622 | 96.54 | 0.46(0.48) | 0.04 | 0.65 | 50.10 | 0.50(0.49) | 0.04 | 0.68 | 50.10 | 0.45(0.48) | 0.04 | 0.64 | 26.19 | * |
| Sb | 10622 | 76.19 | 0.05(0.05) | 0.01 | 0.08 | 4.12 | 0.06(0.05) | 0.01 | 0.08 | 1.42 | 0.05(0.05) | 0.01 | 0.08 | 4.12 | 0.14 |
| TI | 10622 | 99.54 | 0.15(0.10) | 0.01 | 0.18 | 3.04 | 0.17(0.11) | 0.01 | 0.20 | 3.04 | 0.14(0.10) | 0.01 | 0.17 | 2.23 | * |
| Tu | 10622 | 88.60 | 0.07(0.08) | 0.01 | 0.11 | 9.77 | 0.07(0.08) | 0.01 | 0.12 | 5.44 | 0.06(0.07) | 0.01 | 0.11 | 9.77 | 0.11 |
| As | 10622 | 99.21 | 7.22(10.76) | 0.49 | 17.30 | 1441 | 7.62(11.00) | 0.94 | 18.29 | 1189 | 7.12(10.67) | 0.49 | 17.00 | 1441 | 0.19 |

IQR, inter-quartile range; N, number; *, <0.05.

**Table S2**. Associations of multiple blood and urinary metals with SUI risk after adjustment for other metals in the NHANES 2003-2018

| Metals | Q1 | Q2 |  | Q3 |  | Q4 |  | Continuous |  |
| --- | --- | --- | --- | --- | --- | --- | --- | --- | --- |
|  |  | OR (95% CI) | P value | OR (95 % CI) | P value | OR (95 % CI) | P value | OR (95 % CI) | P value |
| **Model 1:** |  |  |  |  |  |  |  |  |  |
| **Blood (μg/L)** |  |  |  |  |  |  |  |  |  |
| Cd | Ref | 1.17 (1.00-1.37) | 0.054 | 1.09 (0.93-1.27) | 0.285 | 1.29 (1.09-1.52) | **0.003** | 1.16 (1.07-1.25) | **<0.001** |
| Pb | Ref | 1.20 (1.03-1.39) | **0.017** | 1.29 (1.10-1.51) | **0.002** | 1.34 (1.12-1.61) | **0.002** | 1.15 (1.04-1.26) | **0.005** |
| Hg | Ref | 1.38 (1.19-1.60) | **<0.001** | 1.27 (1.09-1.48) | **0.002** | 1.27 (1.08-1.49) | **0.004** | 1.08 (1.02-1.14) | **0.012** |
| **Model 2:** |  |  |  |  |  |  |  |  |  |
| **Urine (μg/g creatinine)** |  |  |  |  |  |  |  |  |  |
| Ba | Ref | 0.92 (0.78-1.08) | 0.313 | 0.91 (0.77-1.07) | 0.241 | 0.96 (0.81-1.14) | 0.626 | 0.99 (0.93-1.05) | 0.737 |
| Cd | Ref | 1.26 (1.07-1.50) | **0.007** | 1.50 (1.27-1.78) | **<0.001** | 1.64 (1.37-1.97) | **<0.001** | 1.22 (1.14-1.32) | **<0.001** |
| Co | Ref | 1.06 (0.88-1.26) | 0.543 | 1.10 (0.92-1.32) | 0.280 | 1.08 (0.90-1.30) | 0.409 | 1.01 (0.93-1.10) | 0.817 |
| Cs | Ref | 0.88 (0.74-1.05) | 0.156 | 1.06 (0.88-1.26) | 0.542 | 1.08 (0.89-1.32) | 0.418 | 1.21 (1.06-1.40) | **0.006** |
| Mo | Ref | 0.97 (0.83-1.13) | 0.667 | 0.96 (0.82-1.12) | 0.596 | 0.96 (0.81-1.13) | 0.619 | 0.96 (0.87-1.05) | 0.333 |
| Pb | Ref | 1.36 (1.16-1.60) | **<0.001** | 1.24 (1.04-1.47) | **0.016** | 1.34 (1.11-1.62) | **0.002** | 1.08 (0.98-1.18) | 0.110 |
| Sb | Ref | 1.06 (0.91-1.24) | 0.449 | 0.99 (0.84-1.16) | 0.865 | 1.02 (0.87-1.20) | 0.807 | 1.00 (0.92-1.09) | 0.982 |
| TI | Ref | 0.93 (0.79-1.10) | 0.377 | 0.91 (0.77-1.08) | 0.267 | 0.86 (0.72-1.03) | 0.097 | 0.91 (0.80-1.02) | 0.112 |
| Tu | Ref | 0.96 (0.82-1.12) | 0.572 | 0.93 (0.79-1.08) | 0.334 | 0.85 (0.72-1.01) | 0.058 | 0.97 (0.91-1.04) | 0.366 |
| As | Ref | 1.03 (0.89-1.21) | 0.672 | 1.04 (0.89-1.22) | 0.608 | 1.00 (0.85-1.18) | 0.986 | 0.99 (0.94-1.05) | 0.778 |

Model 1 was adjusted for three blood metals, age, gender, race/ethnicity, education levels, marital status, Poverty Income Ratio, physical activity, body mass index, waist circumference, serum cotinine, alcohol use and NHANES cycles. Model 2 was adjusted for 10 urinary metals, age, gender, race/ethnicity, education levels, marital status, Poverty Income Ratio, physical activity, body mass index, waist circumference, serum cotinine, alcohol use and NHANES cycles. Continuous, Ln-transformed concentration of metal; CI: confidence interval; OR: odds ratio; Q, quartile; Ref, reference.

**Bold**: *p* < 0.05.

**Table S3.** The SUI inclusion probability of single blood and urinary metals in the NHANES 2003-2018 cycles

| Metals | PIP value | | | | |
| --- | --- | --- | --- | --- | --- |
|  | Total | 20 ≤ Age < 60 | Age ≥ 60 | Male | Female |
| Blood (μg/L) |  |  |  |  |  |
| Cd | 1.0000 | 0.9993 | 0.3165 | 0.9289 | 0.9573 |
| Pb | 0.9950 | 1.0000 | 0.3084 | 0.2150 | 0.9450 |
| Hg | 0.9862 | 0.9458 | 0.3893 | 0.0205 | 0.9022 |
| Urine (μg/g creatinine) |  |  |  |  |  |
| Ba | 0.2646 | 0.2588 | 0.2853 | 0.0383 | 0.0045 |
| Cd | 1.0000 | 1.0000 | 0.0376 | 0.0318 | 1.0000 |
| Co | 0.2638 | 0.3537 | 0.1729 | 0.0147 | 0.0000 |
| Cs | 0.9869 | 1.0000 | 0.0103 | 0.0000 | 1.0000 |
| Mo | 0.2395 | 0.4104 | 0.0167 | 0.0112 | 0.0000 |
| Pb | 0.8260 | 0.7173 | 0.0407 | 0.0780 | 0.0479 |
| Sb | 0.1685 | 0.2247 | 0.0881 | 0.0000 | 0.0005 |
| TI | 0.6368 | 0.4508 | 0.0168 | 0.0030 | 0.2663 |
| Tu | 0.1852 | 0.2239 | 0.0220 | 0.0000 | 0.0019 |
| As | 0.6784 | 0.7289 | 0.0223 | 0.0133 | 0.1184 |

PIP, posteriori inclusion probability.


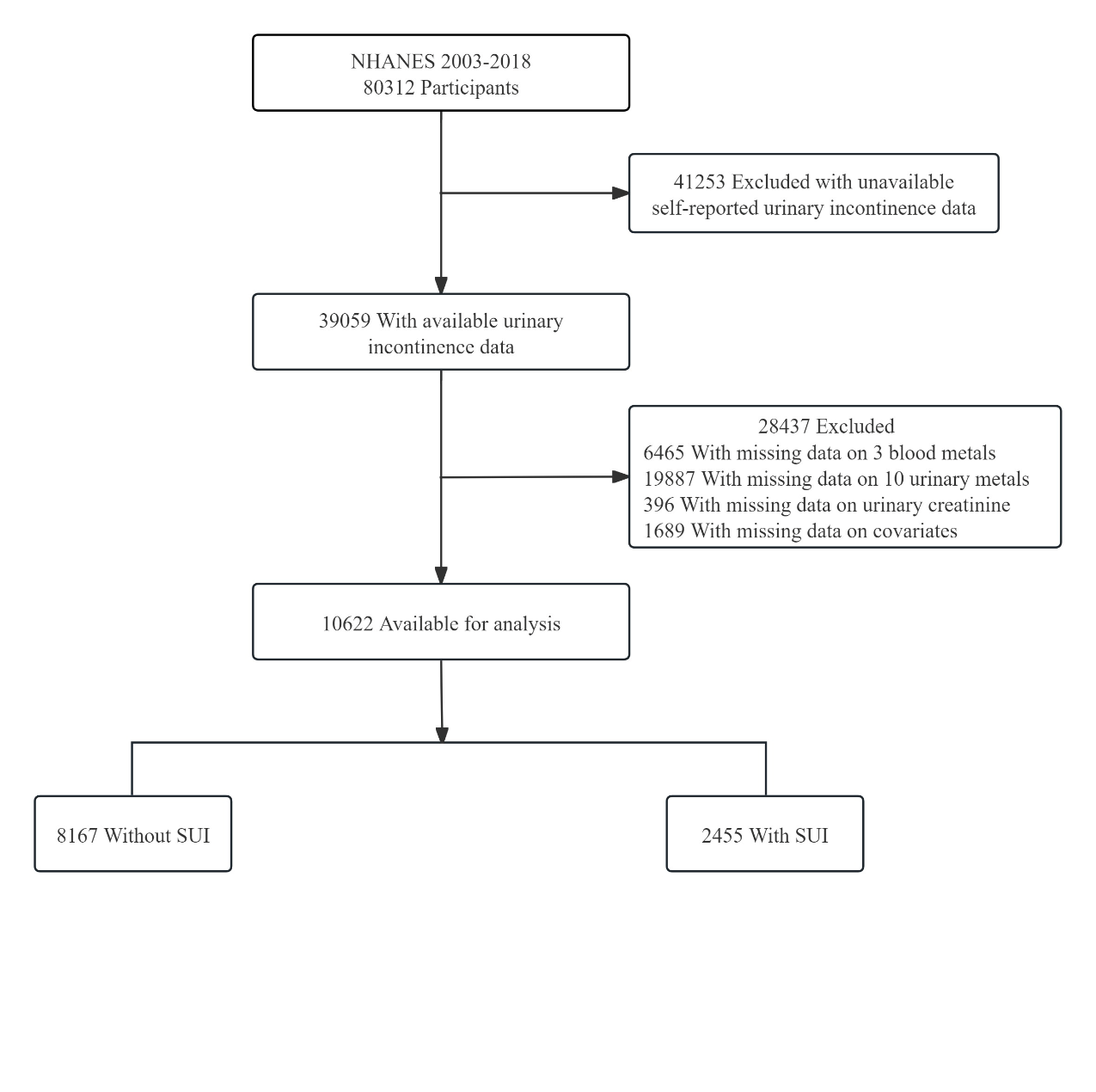


­ Fig. S1 Flow diagram of the screening and enrollment of study participants.


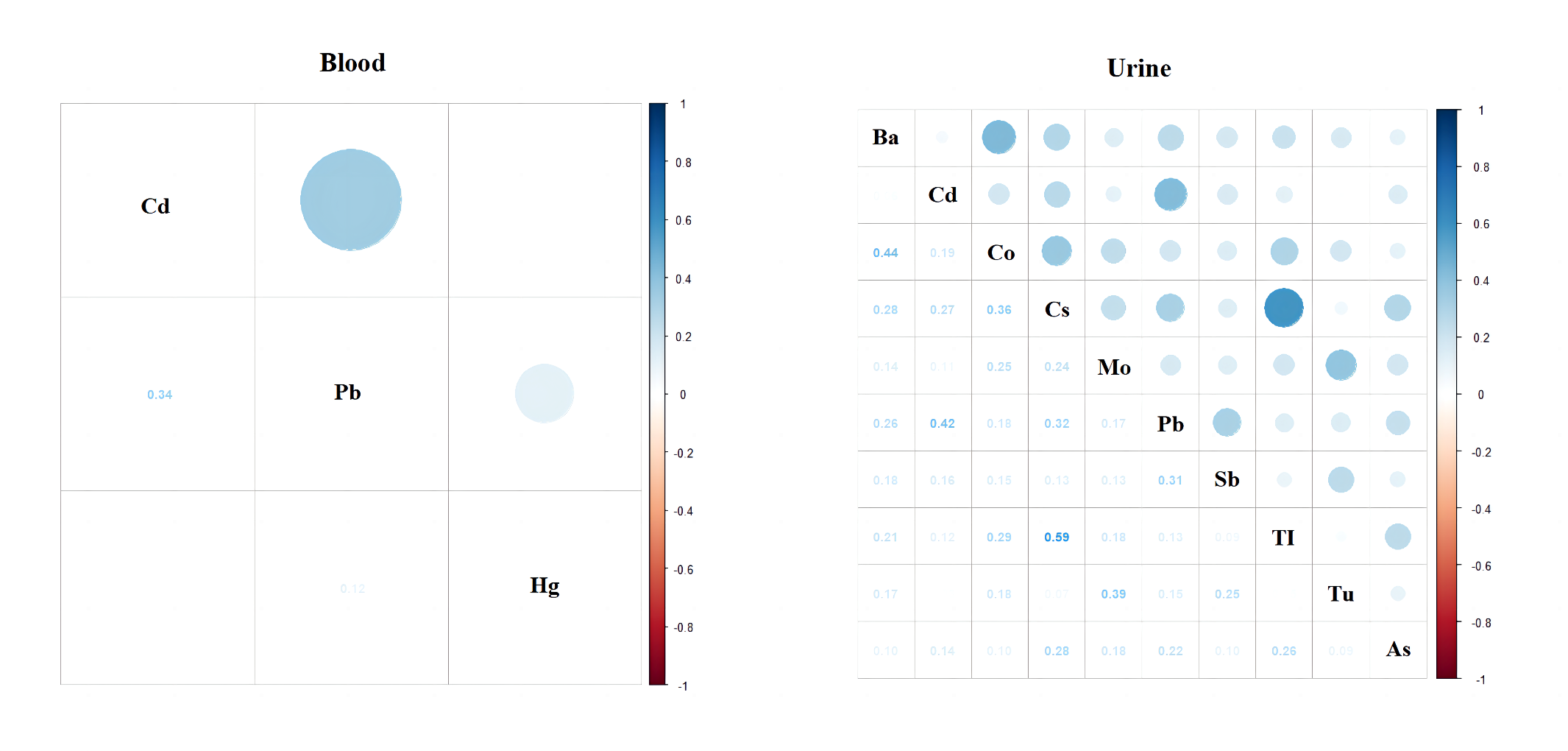


Fig. S2 The Spearman correlation between blood and urinary metals after In-transformed.


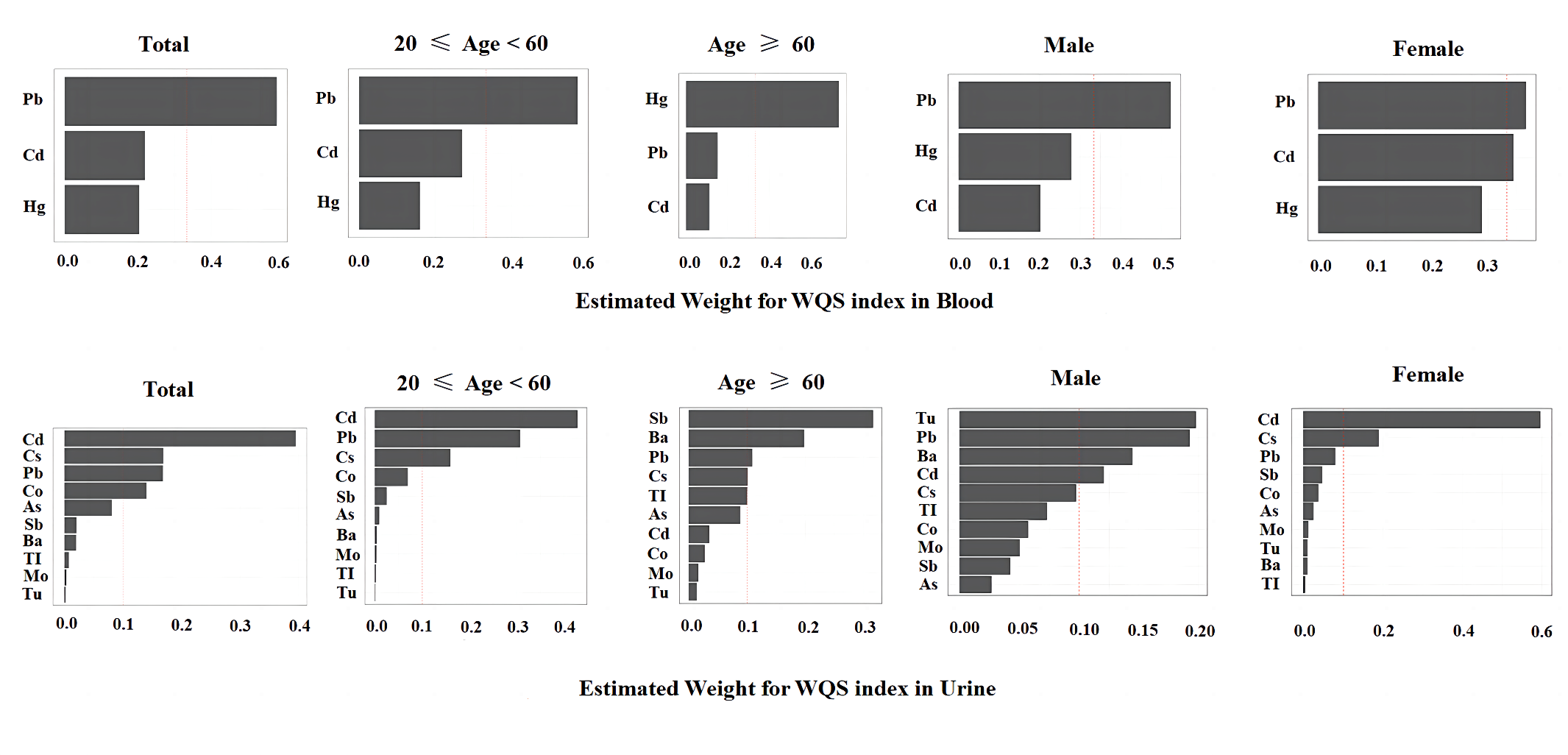


Fig. S3 Estimated weights of blood and urinary metals for SUI by WQS models adjusted for age, gender, race/ethnicity, education levels, marital status, Poverty Income Ratio, physical activity, body mass index, waist circumference, serum cotinine, alcohol use and NHANES cycles.


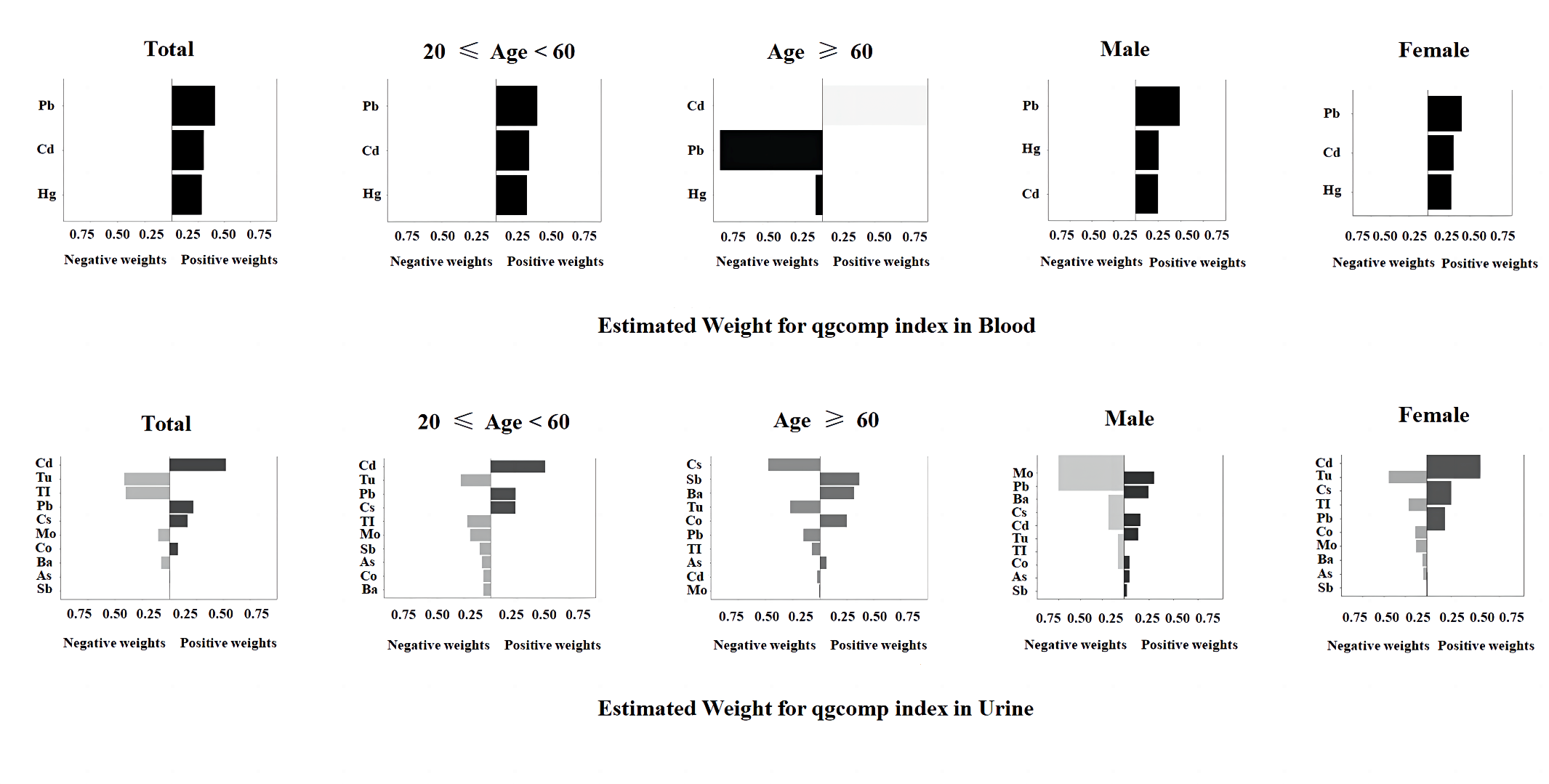


Fig. S4 The positive and negative weights of blood and urinary metals for SUI by qgcomp models adjusted for age, gender, race/ethnicity, education levels, marital status, Poverty Income Ratio, physical activity, body mass index, waist circumference, serum cotinine, alcohol use and NHANES cycles.


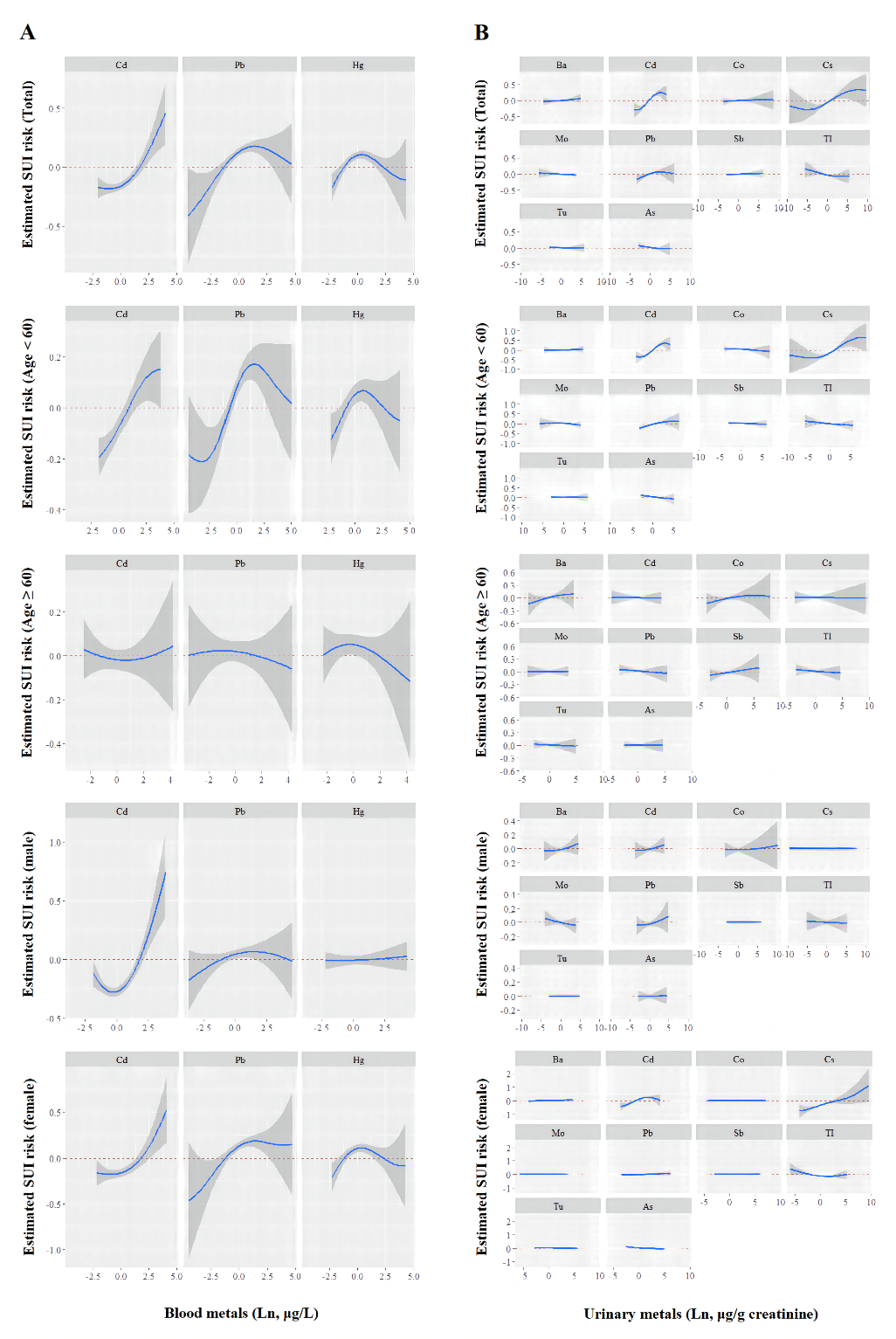
­

Fig. S5 Univariate exposure-response functions for each blood (A) and urinary metals (B) with the other metals fixed at the median. The results were assessed by the Bayesian Kernel Machine Regression (BKMR) models. Models (total) were adjusted for age, gender, race/ethnicity, education levels, marital status, Poverty Income Ratio, physical activity, body mass index, waist circumference, serum cotinine, alcohol use and NHANES cycles.


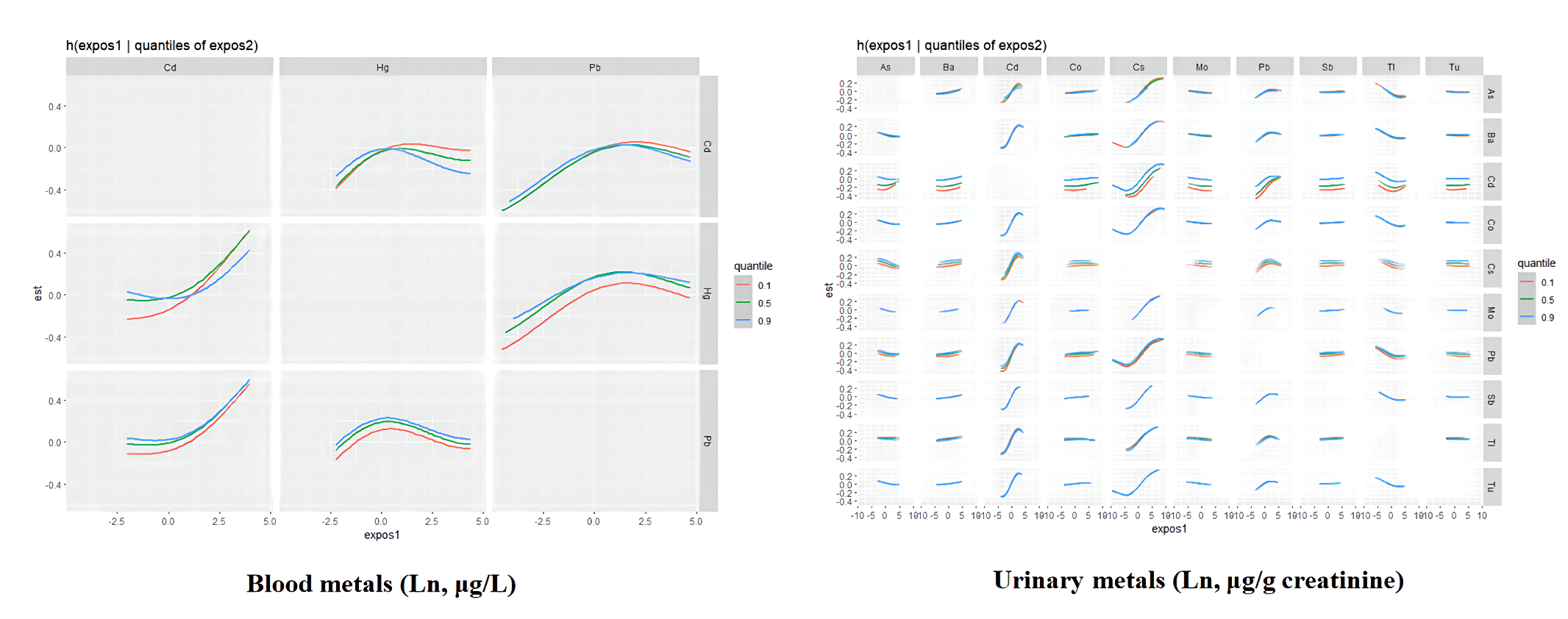


Fig. S6 The interaction of blood and urinary metals for SUI by BKMR models adjusted for age, gender, race/ethnicity, education levels, marital status, Poverty Income Ratio, physical activity, body mass index, waist circumference, serum cotinine, alcohol use and NHANES cycles.


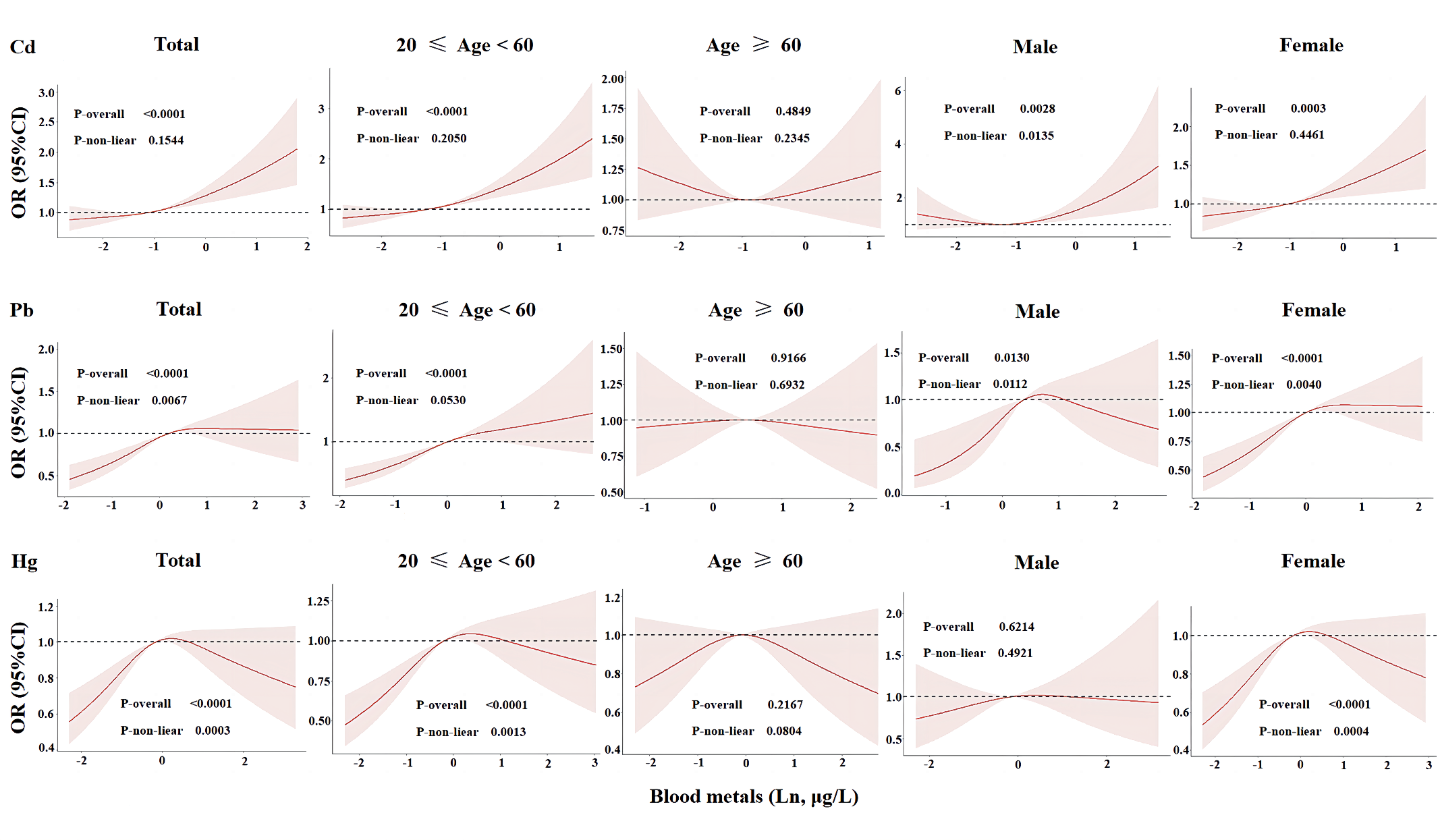


Fig. S7 Dose-response relationship between blood Cd, Pb, Hg and SUI risk was estimated by RCS models in the total population and subgroups. Models were adjusted for age, gender, race/ethnicity, education levels, marital status, Poverty Income Ratio, physical activity, body mass index, waist circumference, serum cotinine, alcohol use and NHANES cycles. Solid line, odds ratios; red-shade, 95 % CI.


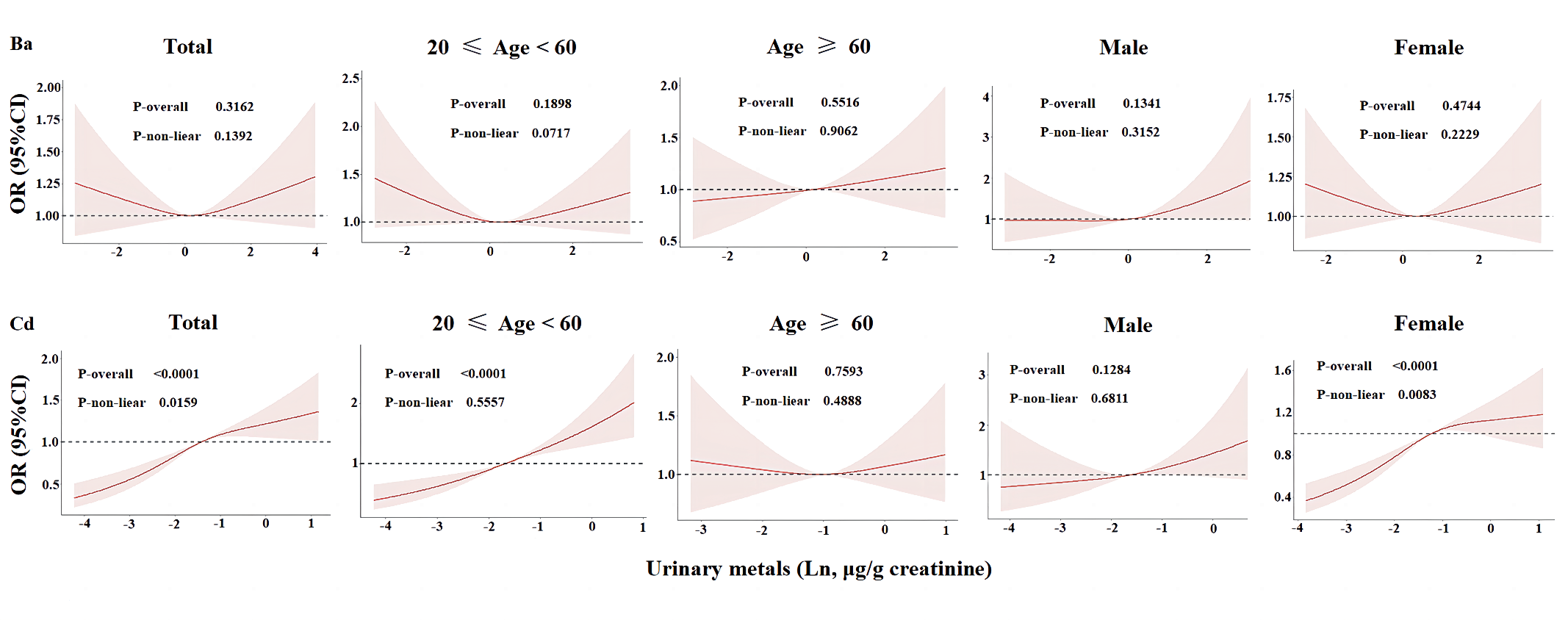
Fig. S8 Dose-response relationship between urinary Ba, Cd, and SUI risk was estimated by RCS models in the total population and subgroups. Models were adjusted for age, gender, race/ethnicity, education levels, marital status, Poverty Income Ratio, physical activity, body mass index, waist circumference, serum cotinine, alcohol use and NHANES cycles. Solid line, odds ratios; red-shade, 95 % CI.


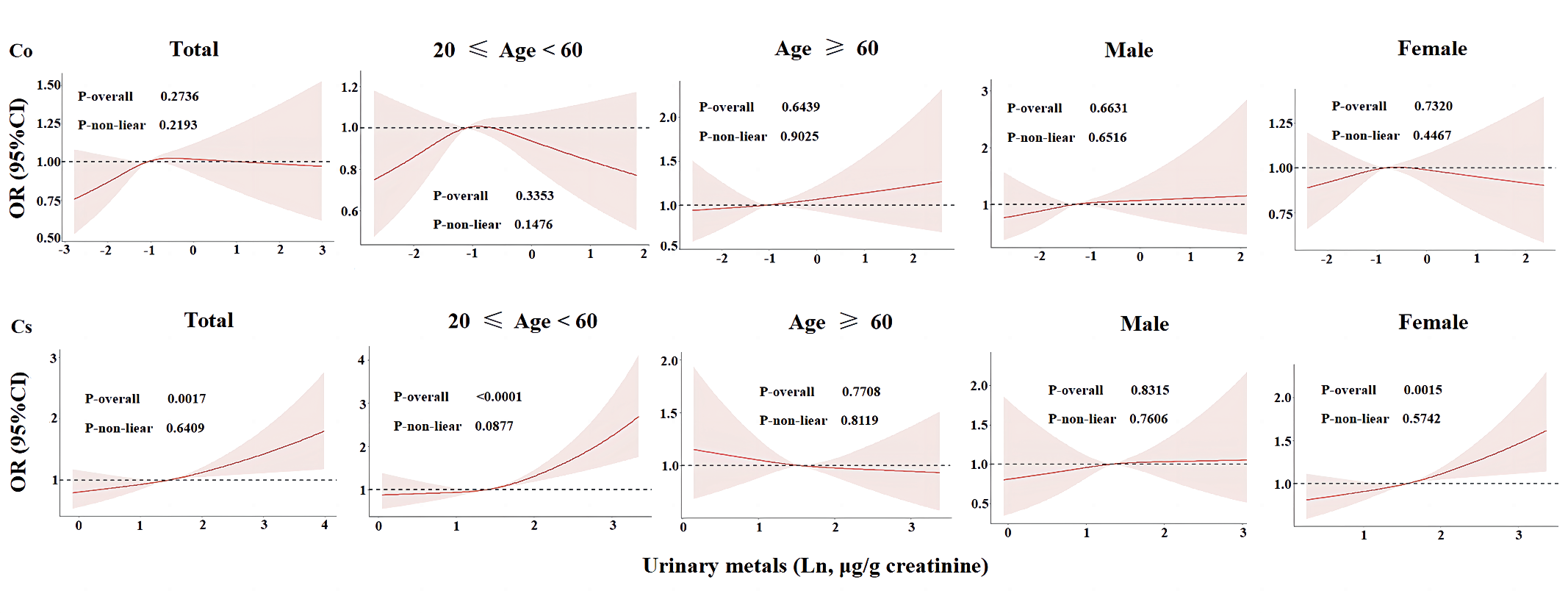
Fig. S9 Dose-response relationship between urinary Co, Cs, and SUI risk was estimated by RCS models in the total population and subgroups. Models were adjusted for age, gender, race/ethnicity, education levels, marital status, Poverty Income Ratio, physical activity, body mass index, waist circumference, serum cotinine, alcohol use and NHANES cycles. Solid line, odds ratios; red-shade, 95 % CI.


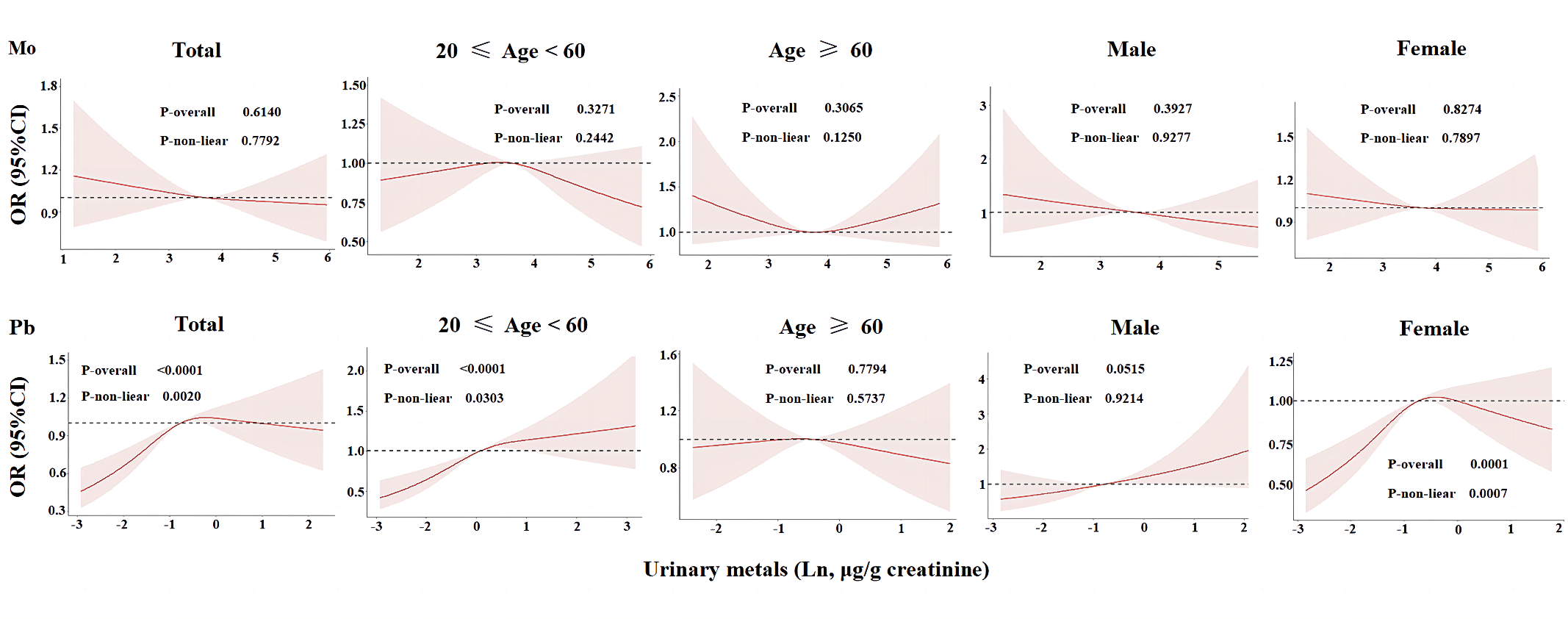
Fig. S10 Dose-response relationship between urinary Mo, Pb, and SUI risk was estimated by RCS models in the total population and subgroups. Models were adjusted for age, gender, race/ethnicity, education levels, marital status, Poverty Income Ratio, physical activity, body mass index, waist circumference, serum cotinine, alcohol use and NHANES cycles. Solid line, odds ratios; red-shade, 95 % CI.


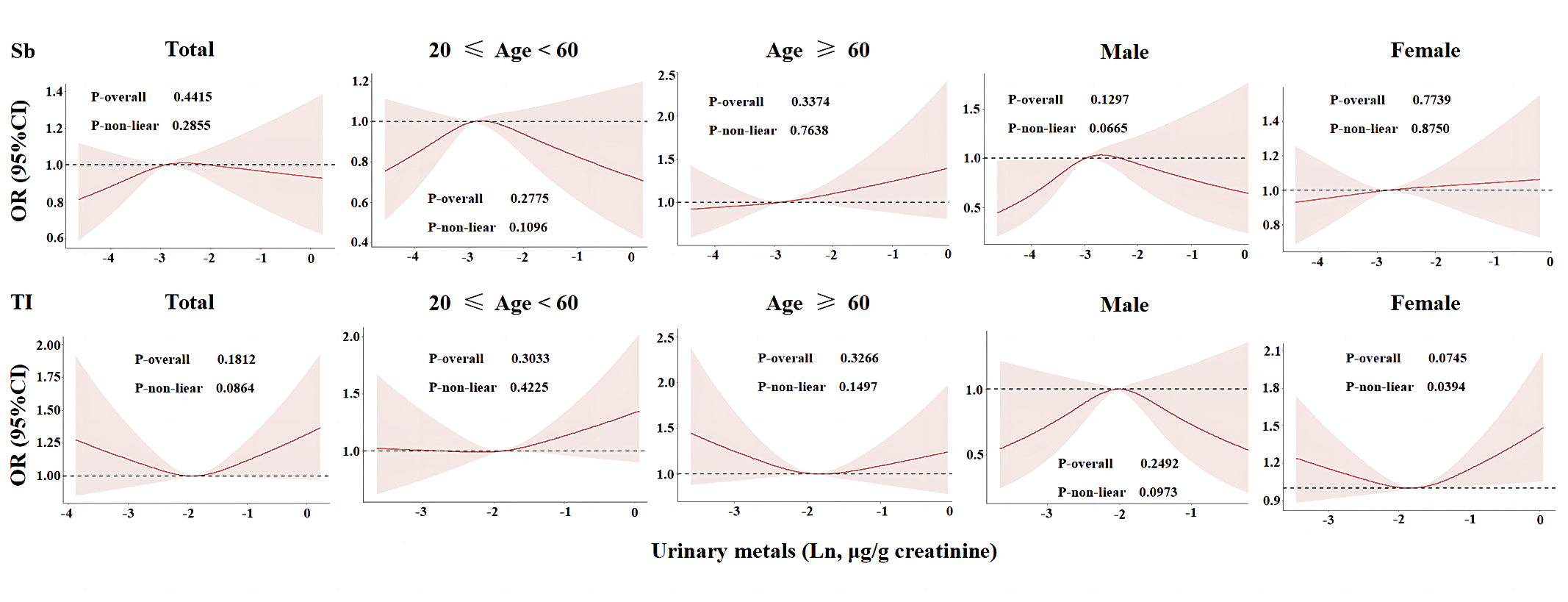
Fig. S11 Dose-response relationship between urinary Sb, TI, and SUI risk was estimated by RCS models in the total population and subgroups. Models were adjusted for age, gender, race/ethnicity, education levels, marital status, Poverty Income Ratio, physical activity, body mass index, waist circumference, serum cotinine, alcohol use and NHANES cycles. Solid line, odds ratios; red-shade, 95 % CI.


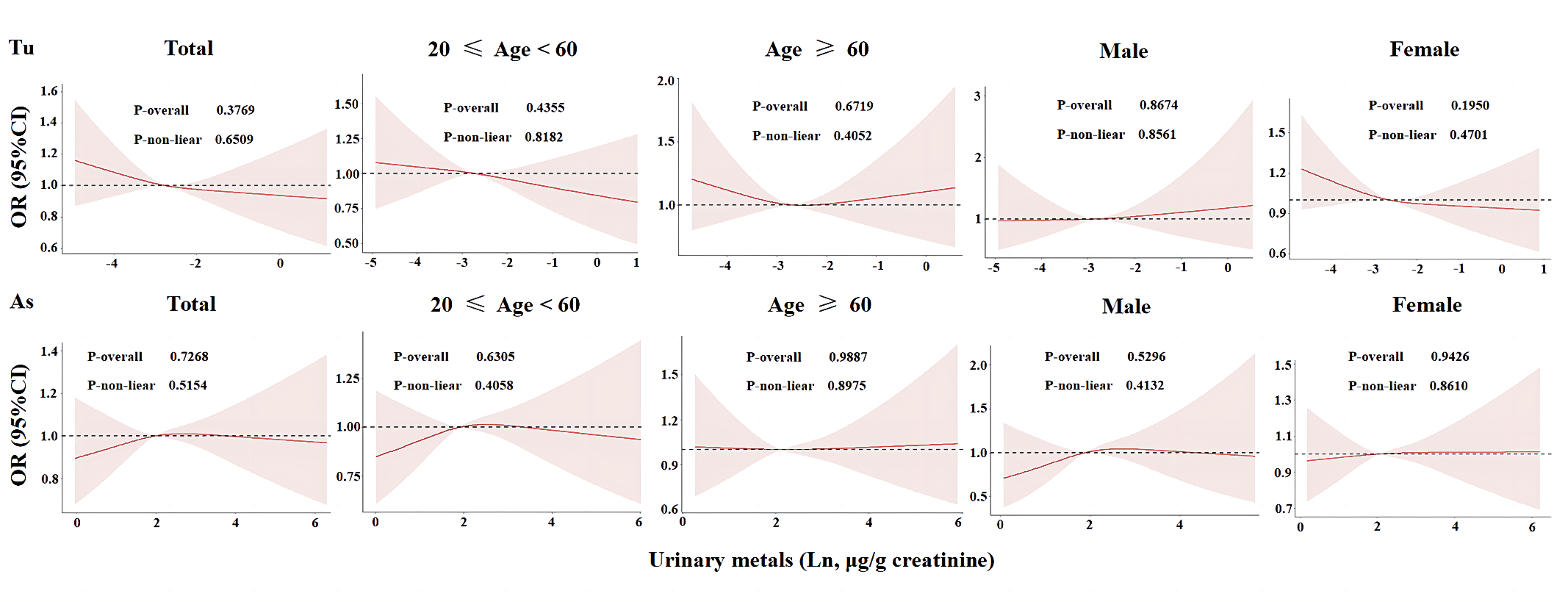
Fig. S12 Dose-response relationship between urinary Tu, As, and SUI risk was estimated by RCS models in the total population and subgroups. Models were adjusted for age, gender, race/ethnicity, education levels, marital status, Poverty Income Ratio, physical activity, body mass index, waist circumference, serum cotinine, alcohol use and NHANES cycles. Solid line, odds ratios; red-shade, 95 % CI.

­­­­
